# Supplementary material for: A Rapid and Inexpensive PCR Test for Mastitis Diagnosis Based on NGS Data
Source: Pathogens. 2024 May 17;13(5):423. doi: 10.3390/pathogens13050423 (PMC11487460; doi:10.3390/pathogens13050423)
Supplement: Supplementary file 1 [file pathogens-13-00423-s001.zip › pathogens-2949471-Supplementary.pdf]

pathogens

MDPI

Supplementary Materials

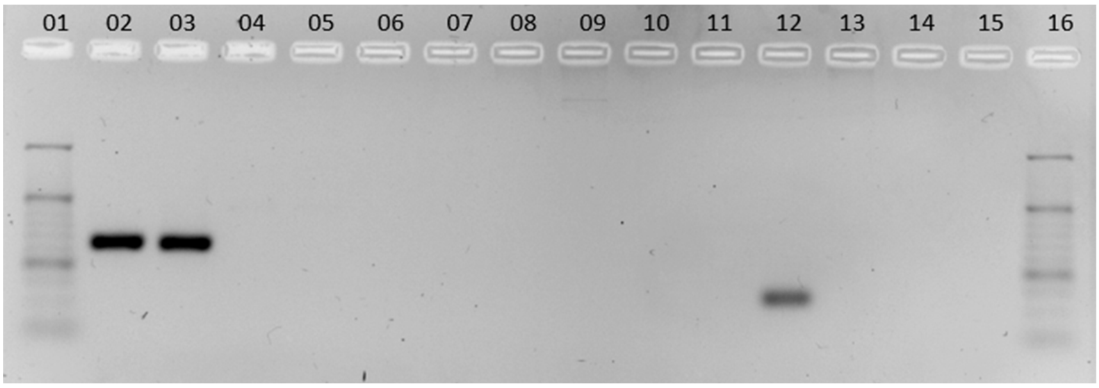

**Sraga1**	-	-	-	-	-	-	-	-	-	-	+	-	-	-
**Srdys1**	-	-	-	-	-	-	-	-	-	-	-	-	-	-
**Srube3**	+	+	-	-	-	-	-	-	-	-	-	-	-	-
**Staur3**	-	-	-	-	-	-	-	-	-	-	-	-	-	-

Fig. S1. Multiplex PCR results. Lane 01, 50 bp DNA ladder (with the following distribution – 50, 100,150, 200, 250, 300, 400, 500, 600, 700, 800, 900, 1000); Lane 02, *Streptococcus uberis* 105PP2021; Lane 03, *Streptococcus uberis* 112PP2021; Lane 04, *Aerococcus viridans* 3 006PP2021; Lane 05, *Aerococcus viridans* 2 007PP2021; Lane 06, *Aerococcus viridans* 3 008PP2021; Lane 07, *Enterococcus faecium* 003PP2021; Lane 08, *Enterococcus faecium* 004PP2021; Lane 09, *Enterococcus faecalis* 022PP2021; Lane 10, *Staphylococcus epidermidis* 001PP2022; Lane 11, *Staphylococcus sciuri* 001PP2020; Lane 12, *Staphylococcus sciuri* 002PP2021; Lane 13, *Staphylococcus sciuri* 003PP2021; Lane 14, *Staphylococcus xylosus* 001PP2021; Lane 15, *Staphylococcus xylosus* 002PP2022; Lane 16, 50 bp DNA ladder.

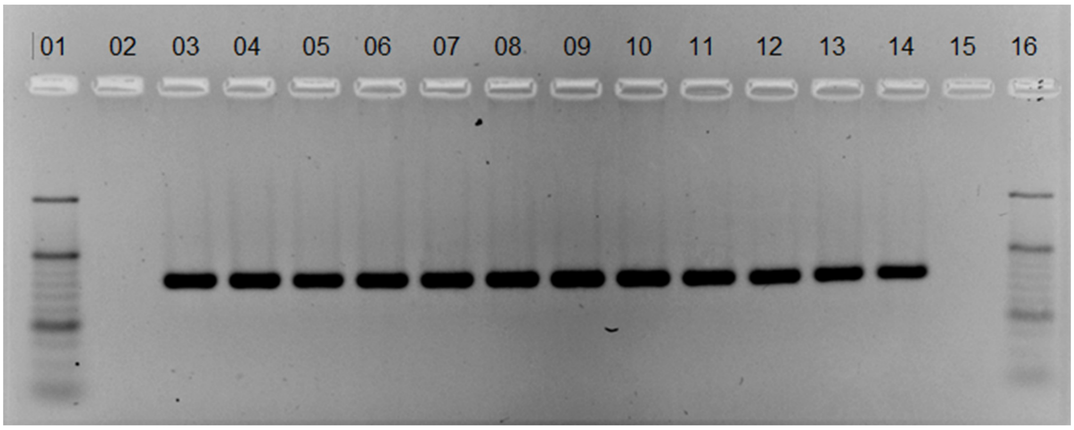

**Sraga1**	-	-	-	-	-	-	-	-	-	-	-	-	-	-
**Srdys1**	-	-	-	-	-	-	-	-	-	-	-	-	-	-
**Srube3**	-	-	-	-	-	-	-	-	-	-	-	-	-	-
**Staur3**	-	+	+	+	+	+	+	+	+	+	+	+	+	-

Figure S2. Multiplex PCR results. Lane 01, 50 bp DNA ladder (with the following distribution – 50, 100,150, 200, 250, 300, 400, 500, 600, 700, 800, 900, 1000); Lane 02, *Staphylococcus xylosus* 003PP2022; Lane 03, *Staphylococcus aureus* ATCC 6538P Rosenbach; Lane 04, *Staphylococcus aureus* PCM 2267; Lane 05, *Staphylococcus aureus* PCM 458/2195; Lane 06, *Staphylococcus aureus* PCM 2101; Lane 07, *Staphylococcus aureus* PCM 2054; Lane 08, *Staphylococcus aureus* PCM 1650; Lane 09, *Staphylococcus aureus* PCM 1116; Lane 10, *Staphylococcus aureus* PCM 1115; Lane 11, *Staphylococcus aureus* PCM 1102;

Lane 12, *Staphylococcus aureus* PCM 565; Lane 13, *Staphylococcus aureus* PCM 502; Lane 14, *Staphylococcus aureus* PCM 1937; Lane 15, NTC; Lane 16, 50 bp DNA ladder.

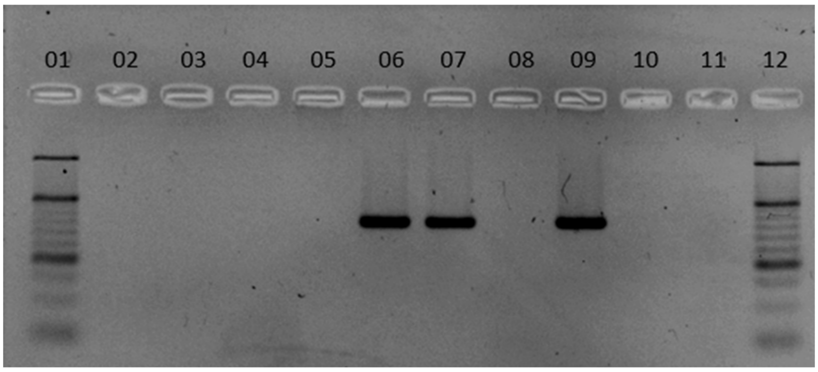

**Sraga1**	-	-	-	-	-	-	-	-	-	-
**Srdys1**	-	-	-	-	-	-	-	-	-	-
**Srube3**	-	-	-	-	-	-	-	-	-	-
**Staur3**	-	-	-	-	+	+	-	+	-	-

Figure S3. Multiplex PCR results. Lane 01, 50 bp DNA ladder (with the following distribution – 50, 100,150, 200, 250, 300, 400, 500, 600, 700, 800, 900, 1000); Lane 02, *Enterococcus* 009PP2022; Lane 03, *Enterococcus* 010PP2022; Lane 04, *Enterococcus* 012PP2022; Lane 05, *Enterococcus* 013PP2022; Lane 06, *Staphylococcus* 008PP2022; Lane 07, *Staphylococcus* 009PP2022; Lane 08, *Staphylococcus* 010PP2022; Lane 09, *Staphylococcus* 011PP2022; Lane 10, *Staphylococcus* 012PP2022; Lane 11, NTC; Lane 12, 50 bp DNA ladder.

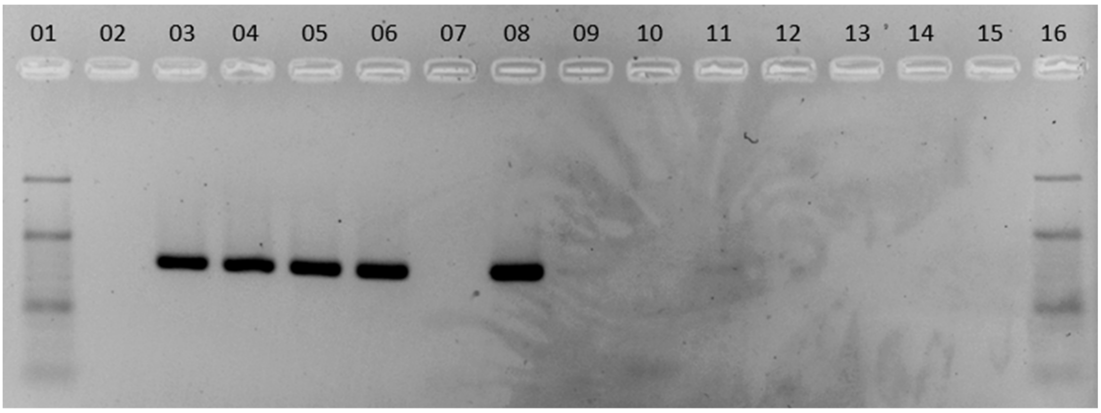

**Sraga1**	-	-	-	-	-	-	-	-	-	-	-	-	-	-
**Srdys1**	-	-	-	-	-	-	-	-	-	-	-	-	-	-
**Srube3**	-	-	-	-	-	-	-	-	-	-	-	-	-	-
**Staur3**	-	+	+	+	+	-	+	+	-	+	-	-	-	-

Figure S4. Multiplex PCR results. Lane 01, 50 bp DNA ladder (with the following distribution – 50, 100,150, 200, 250, 300, 400, 500, 600, 700, 800, 900, 1000); Lane 02, *Enterococcus* 011PP2022; Lane 03, *Staphylococcus* 013PP2022; Lane 04, *Staphylococcus* 014PP2022; Lane 05, *Staphylococcus* 015PP2022; Lane 06, *Staphylococcus* 016PP2022; Lane 07, *Staphylococcus* 017PP2022; Lane 08, *Staphylococcus* 018PP2022; Lane 09, *Staphylococcus* 019PP2022; Lane 10, *Staphylococcus* 023PP2022; Lane 11, *Staphylococcus* 025PP2022; Lane 12, *Staphylococcus* 026PP2022; Lane 13, *Staphylococcus* 027PP2022; Lane 14, *Staphylococcus* 030PP2022; Lane 15, *Staphylococcus* 031PP2022; Lane 16, 50 bp DNA ladder.

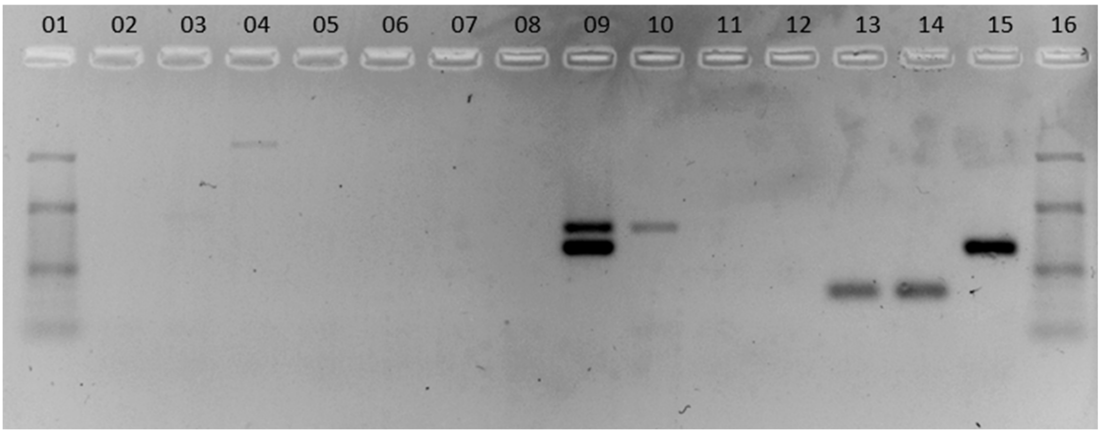

**Sraga1**	-	-	-	-	-	-	-	-	-	-	-	+	+	-
**Srdys1**	-	-	-	-	-	-	-	-	-	-	-	-	-	-
**Srube3**	-	-	-	-	-	-	-	+	-	-	-	-	-	+
**Staur3**	-	-	-	-	-	-	-	+	+	-	-	-	-	-

Figure S5. Multiplex PCR results. Lane 01, 50 bp DNA ladder (with the following distribution – 50, 100,150, 200, 250, 300, 400, 500, 600, 700, 800, 900, 1000); Lane 02, *Staphylococcus* 032PP2022; Lane 03, *Staphylococcus* 034PP2022; Lane 04, *Staphylococcus* 038PP2022; Lane 05, *Staphylococcus* 039PP2022; Lane 06, *Staphylococcus* 040PP2022; Lane 07, *Staphylococcus* 041PP2022; Lane 08, *Staphylococcus* 042PP2022; Lane 09, *Staphylococcus* 043PP2022; Lane 10, *Staphylococcus* 044PP2022; Lane 11, *Staphylococcus* 045PP2022; Lane 12, *Staphylococcus* 046PP2022; Lane 13, *Streptococcus agalactiae* 021PP2018; Lane 14, *Streptococcus agalactiae* 022PP2021; Lane 15, *Streptococcus* 003PP2017; Lane 16, 50 bp DNA ladder.

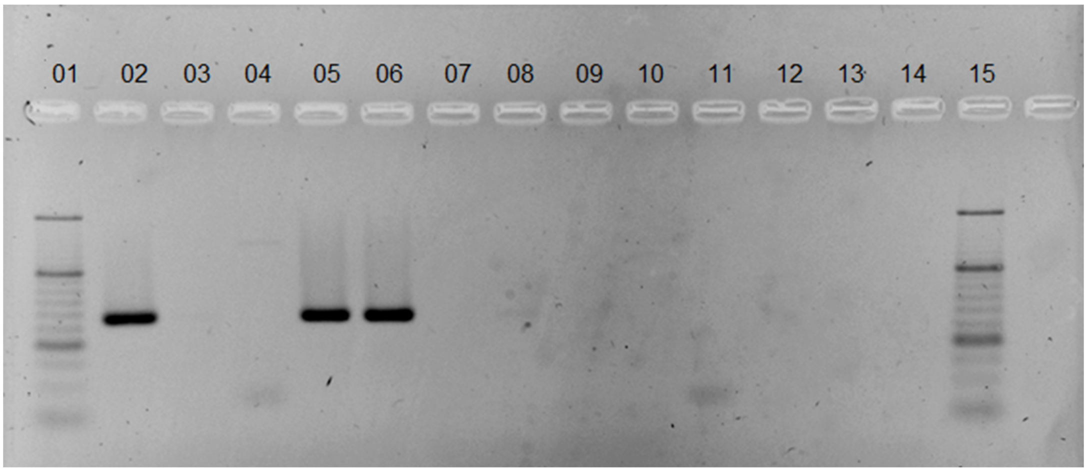

**Sraga1**	-	-	-	-	-	-	-	-	-	-	-	-	-
**Srdys1**	-	-	+	-	-	-	-	-	-	+	-	-	-
**Srube3**	+	-	-	+	+	-	-	-	-	-	-	-	-
**Staur3**	-	-	-	-	-	-	-	-	-	-	-	-	-

Figure S6. Multiplex PCR results. Lane 01, 50 bp DNA ladder (with the following distribution – 50, 100,150, 200, 250, 300, 400, 500, 600, 700, 800, 900, 1000); Lane 02, *Streptococcus* 004PP2017; Lane 03, *Streptococcus* 006PP2017; Lane 04, *Streptococcus* 008PP2018; Lane 05, *Streptococcus* 009PP2019; Lane 06, *Streptococcus* 014PP2022; Lane 07, *Streptococcus* 017PP2022; Lane 08, *Streptococcus* 018PP2022; Lane 09, *Staphylococcus warneri* ATCC 27836; Lane 10, *Staphylococcus epidermidis* ATCC 14990; Lane 11, *Streptococcus dysgalactiae* ATCC 12394; Lane 12, *Aerococcus viridans* ATCC 11563; Lane 13, *Enterococcus faecalis* ATCC 29212; Lane 14, NTC; Lane 15, 50 bp DNA ladder.

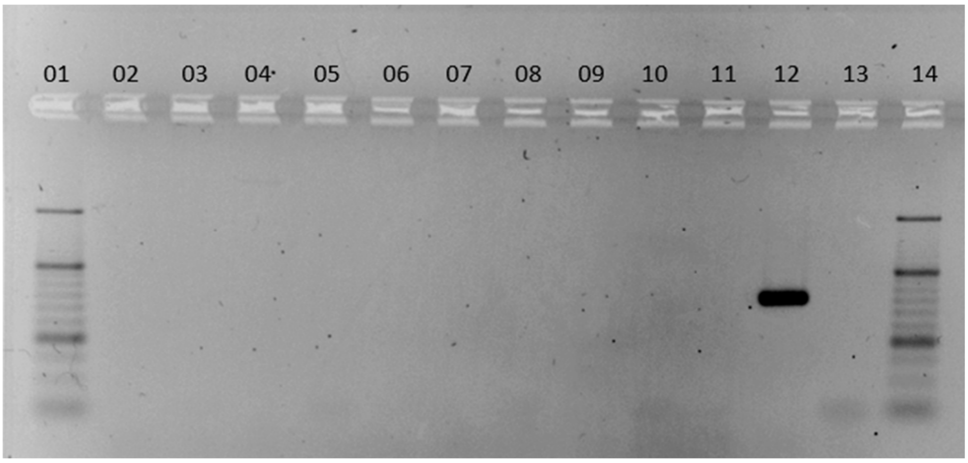

**Sraga1**	-	-	-	-	-	-	-	-	-	-	-	-
**Srdys1**	-	-	-	-	-	-	-	-	-	-	-	-
**Srube3**	-	-	-	-	-	-	-	-	-	-	-	-
**Staur3**	-	-	-	-	-	-	-	-	-	-	+	-

Figure S7. Multiplex PCR results. Lane 01, 50 bp DNA ladder (with the following distribution – 50, 100,150, 200, 250, 300, 400, 500, 600, 700, 800, 900, 1000); Lane 02, *Staphylococcus* 047PP2022; Lane 03, *Staphylococcus* 048PP2022; Lane 04, *Staphylococcus* 049PP2022; Lane 05, *Staphylococcus* 050PP2022; Lane 06, *Staphylococcus* 051PP2022; Lane 07, *Staphylococcus* 052PP2022; Lane 08, *Staphylococcus* 053PP2022; Lane 09, *Staphylococcus* 054PP2022; Lane 10, *Staphylococcus* 055PP2022; Lane 11, *Staphylococcus* 056PP2022; Lane 12, *Staphylococcus* 057PP2022; Lane 13, NTC; Lane 14, 50 bp DNA ladder.

Table S1. Bacterial genomes assemblies used for test designing as true positives to detect conservative core sequence for analyzed species. .

True Positive Streptococcus uberis	*True Positive Streptococcus dysgalactiae*	*True Positive Staphylococcus aureus*	*True Positive Streptococcus agalactiae*
GCF_000975325.1	*GCF_000307185.1*	*GCF_900004855.1*	*GCF_000007265.1*
GCF_000975335.1	*GCF_000317855.1*	*GCF_900017485.1*	*GCF_000012705.1*
GCF_000975345.1	*GCF_000970985.1*	*GCF_900017495.1*	*GCF_000167755.1*
GCF_000975355.1	*GCF_000971005.1*	*GCF_900017505.1*	*GCF_000186445.1*
GCF_000975405.1	*GCF_001038445.1*	*GCF_900017515.1*	*GCF_000196055.1*
GCF_000975415.1	*GCF_001072165.1*	*GCF_900017525.1*	*GCF_000264975.1*
GCF_000975435.1	*GCF_001682735.1*	*GCF_900017535.1*	*GCF_000287955.1*
GCF_000975445.1	*GCF_001682745.1*	*GCF_900017545.1*	*GCF_000287975.1*
GCF_000975485.1	*GCF_001682755.1*	*GCF_900017555.1*	*GCF_000287995.1*
GCF_000975495.1	*GCF_001682765.1*	*GCF_900017565.1*	*GCF_000288015.1*
GCF_000975505.1	*GCF_001682815.1*	*GCF_900017575.1*	*GCF_000288035.1*
GCF_000975545.1	*GCF_001682825.1*	*GCF_900017585.1*	*GCF_000288055.1*
GCF_002814135.1	*GCF_001704435.1*	*GCF_900017595.1*	*GCF_000288075.1*
GCF_009706695.1	*GCF_001704445.1*	*GCF_900017605.1*	*GCF_000288095.1*
GCF_009706705.1	*GCF_001704455.1*	*GCF_900017615.1*	*GCF_000288115.1*

GCF_009706755.1	*GCF_001704515.1*	*GCF_900017625.1*	*GCF_000288135.1*
GCF_009706785.1	*GCF_001704525.1*	*GCF_900017635.1*	*GCF_000288155.1*
GCF_009706805.1	*GCF_002094215.1*	*GCF_900017645.1*	*GCF_000288175.1*
GCF_009706815.1	*GCF_003202195.1*	*GCF_900017655.1*	*GCF_000288195.1*
GCF_009706825.1	*GCF_003967135.1*	*GCF_900017665.1*	*GCF_000288215.1*
GCF_009706845.1	*GCF_008121775.1*	*GCF_900017675.1*	*GCF_000288235.1*
GCF_009706885.1	*GCF_008121785.1*	*GCF_900017685.1*	*GCF_000288255.1*
GCF_009706895.1	*GCF_008121795.1*	*GCF_900017695.1*	*GCF_000288275.1*
GCF_009706905.1	*GCF_008121855.1*	*GCF_900017705.1*	*GCF_000288295.1*
GCF_009706935.1	*GCF_008693725.1*	*GCF_900017715.1*	*GCF_000288315.1*
GCF_009706955.1	*GCF_009380195.1*	*GCF_900017775.1*	*GCF_000288335.1*
GCF_009706985.1	*GCF_009389575.1*	*GCF_900017965.1*	*GCF_000288355.1*
GCF_009706995.1	*GCF_009389595.1*	*GCF_900017975.1*	*GCF_000288375.1*
GCF_009707015.1	*GCF_009497975.1*	*GCF_900017985.1*	*GCF_000288395.1*
GCF_009707035.1	*GCF_009650235.1*	*GCF_900017995.1*	*GCF_000288415.1*
GCF_009707045.1	*GCF_009650255.1*	*GCF_900018005.1*	*GCF_000288435.1*
GCF_009707085.1	*GCF_009650275.1*	*GCF_900018015.1*	*GCF_000288455.1*
GCF_009707105.1	*GCF_009650295.1*	*GCF_900018025.1*	*GCF_000288475.1*
GCF_009807055.1	*GCF_009730735.1*	*GCF_900018035.1*	*GCF_000288495.1*
GCF_016113545.1	*GCF_009730755.1*	*GCF_900018045.1*	*GCF_000288515.1*
GCF_016837285.1	*GCF_009730775.1*	*GCF_900018055.1*	*GCF_000288535.1*
GCF_016837305.1	*GCF_009730795.1*	*GCF_900018065.1*	*GCF_000288555.1*
GCF_016837325.1	*GCF_009730815.1*	*GCF_900018075.1*	*GCF_000288575.1*
GCF_016837345.1	*GCF_009730835.1*	*GCF_900018085.1*	*GCF_000288595.1*
GCF_016837365.1	*GCF_009730855.1*	*GCF_900018095.1*	*GCF_000288615.1*
GCF_016837385.1	*GCF_009730875.1*	*GCF_900018105.1*	*GCF_000288635.1*
GCF_016837405.1	*GCF_009730895.1*	*GCF_900018115.1*	*GCF_000288655.1*
GCF_016837425.1	*GCF_009847065.1*	*GCF_900018125.1*	*GCF_000288675.1*
GCF_016837445.1	*GCF_009847485.1*	*GCF_900018135.1*	*GCF_000288695.1*
GCF_016837455.1	*GCF_009847535.1*	*GCF_900018145.1*	*GCF_000288715.1*
GCF_016837485.1	*GCF_009847585.1*	*GCF_900018155.1*	*GCF_000288735.1*
GCF_016837505.1	*GCF_009847965.1*	*GCF_900018165.1*	*GCF_000288755.1*
GCF_016837525.1	*GCF_009848215.1*	*GCF_900018175.1*	*GCF_000288775.1*
GCF_016837545.1	*GCF_011327345.1*	*GCF_900018185.1*	*GCF_000288795.1*
GCF_016837565.1	*GCF_011379515.1*	*GCF_900018195.1*	*GCF_000288815.1*
GCF_016837585.1	*GCF_011379535.1*	*GCF_900018205.1*	*GCF_000288855.1*
GCF_016838345.1	*GCF_011379545.1*	*GCF_900018215.1*	*GCF_000288875.1*
GCF_016838365.1	*GCF_011379555.1*	*GCF_900018225.1*	*GCF_000288895.1*
GCF_016838375.1	*GCF_011379605.1*	*GCF_900018235.1*	*GCF_000288915.1*

GCF_016838425.1	*GCF_011379625.1*	*GCF_900018245.1*	*GCF_000288935.1*
GCF_016838445.1	*GCF_011379645.1*	*GCF_900018255.1*	*GCF_000288955.1*
GCF_016838465.1	*GCF_011379655.1*	*GCF_900018265.1*	*GCF_000288975.1*
GCF_016838485.1	*GCF_011379705.1*	*GCF_900018275.1*	*GCF_000288995.1*
GCF_016838495.1	*GCF_011379715.1*	*GCF_900018285.1*	*GCF_000289015.1*
GCF_016838525.1	*GCF_011379725.1*	*GCF_900018295.1*	*GCF_000289035.1*
GCF_016838545.1	*GCF_011379745.1*	*GCF_900018305.1*	*GCF_000289055.1*
GCF_016838555.1	*GCF_011379765.1*	*GCF_900018315.1*	*GCF_000289075.1*
GCF_019794295.1	*GCF_011379815.1*	*GCF_900018325.1*	*GCF_000289095.1*
GCF_023060755.1	*GCF_011379825.1*	*GCF_900018685.1*	*GCF_000289155.1*
GCF_023060805.1	*GCF_011379845.1*	*GCF_900018695.1*	*GCF_000289175.1*
GCF_023060825.1	*GCF_012844385.1*	*GCF_900018705.1*	*GCF_000289195.1*
GCF_023060855.1	*GCF_012844405.1*	*GCF_900018715.1*	*GCF_000289215.1*
GCF_023060875.1	*GCF_013004125.1*	*GCF_900018725.1*	*GCF_000289235.1*
GCF_023061875.1	*GCF_014192695.1*	*GCF_900018735.1*	*GCF_000289255.1*
GCF_023061955.1	*GCF_014192895.1*	*GCF_900018745.1*	*GCF_000289275.1*
GCF_023062005.1	*GCF_014893835.1*	*GCF_900018755.1*	*GCF_000289315.1*
GCF_023062295.1	*GCF_016128095.1*	*GCF_900018765.1*	*GCF_000289335.1*
GCF_023062355.1	*GCF_016128135.1*	*GCF_900018775.1*	*GCF_000289355.1*
GCF_023062395.1	*GCF_016724885.1*	*GCF_900019035.1*	*GCF_000289375.1*
GCF_023062425.1	*GCF_016766775.1*	*GCF_900019045.1*	*GCF_000289395.1*
GCF_023062475.1	*GCF_016888305.1*	*GCF_900019055.1*	*GCF_000289415.1*
GCF_023062515.1	*GCF_016888325.1*	*GCF_900019065.1*	*GCF_000289435.1*
GCF_023062555.1	*GCF_016888365.1*	*GCF_900019075.1*	*GCF_000289455.1*
GCF_023062565.1	*GCF_016888375.1*	*GCF_900019085.1*	*GCF_000289475.1*
GCF_023504065.1	*GCF_019265245.1*	*GCF_900019095.1*	*GCF_000289495.1*
GCF_023504085.1	*GCF_019265265.1*	*GCF_900019105.1*	*GCF_000289515.1*
GCF_023504105.1	*GCF_019265285.1*	*GCF_900019115.1*	*GCF_000289535.1*
GCF_023504115.1	*GCF_019265305.1*	*GCF_900019125.1*	*GCF_000289555.1*
GCF_023504135.1	*GCF_019265325.1*	*GCF_900019135.1*	*GCF_000289575.1*
GCF_023504165.1	*GCF_019273775.1*	*GCF_900019145.1*	*GCF_000289595.1*
GCF_023504175.1	*GCF_019856435.1*	*GCF_900019155.1*	*GCF_000289615.1*
GCF_023504185.1	*GCF_020466615.1*	*GCF_900019165.1*	*GCF_000289635.1*
GCF_023504215.1	*GCF_020466705.1*	*GCF_900019175.1*	*GCF_000289655.1*
GCF_023504225.1	*GCF_020466715.1*	*GCF_900019185.1*	*GCF_000289675.1*
GCF_023504265.1	*GCF_020466765.1*	*GCF_900019195.1*	*GCF_000289695.1*
GCF_023504285.1	*GCF_020466775.1*	*GCF_900019205.1*	*GCF_000289715.1*
GCF_023504295.1	*GCF_020466785.1*	*GCF_900020205.1*	*GCF_000289735.1*
GCF_023504325.1	*GCF_020466825.1*	*GCF_900020215.1*	*GCF_000289755.1*

GCF_023504335.1	GCF_020466835.1	GCF_900020225.1	GCF_000289775.1
GCF_023504365.1	GCF_020466865.1	GCF_900020235.1	GCF_000289795.1
GCF_023504385.1	GCF_020466885.1	GCF_900020265.1	GCF_000289815.1
GCF_023504395.1	GCF_020466905.1	GCF_900020275.1	GCF_000289835.1
GCF_023504405.1	GCF_020466925.1	GCF_900020285.1	GCF_000289855.1
GCF_023504445.1	GCF_022532125.1	GCF_900020295.1	GCF_000289875.1
GCF_023504465.1	GCF_022760485.1	GCF_900020305.1	GCF_000289895.1
GCF_023504495.1	GCF_022760515.1	GCF_900020315.1	GCF_000289915.1
GCF_023507605.1	GCF_022760555.1	GCF_900020325.1	GCF_000289935.1
GCF_023507665.1	GCF_022760565.1	GCF_900020335.1	GCF_000289955.1
GCF_900459985.1	GCF_023500005.1	GCF_900020345.1	GCF_000289975.1
GCF_900460135.1	GCF_900459065.1	GCF_900020355.1	GCF_000289995.1
GCF_900475595.1	GCF_900459095.1	GCF_900020365.1	GCF_000290015.1
GCF_900475695.1	GCF_900459135.1	GCF_900020375.1	GCF_000290035.1
	GCF_900459205.1	GCF_900020385.1	GCF_000290055.1
	GCF_900459225.1	GCF_900020395.1	GCF_000290075.1
	GCF_900460145.1	GCF_900020405.1	GCF_000290095.1
	GCF_900460375.1	GCF_900020415.1	GCF_000290115.1
	GCF_900474785.1	GCF_900020425.1	GCF_000290135.1
	GCF_900475145.1	GCF_900020435.1	GCF_000290155.1
	GCF_900475185.1	GCF_900020445.1	GCF_000290175.1
	GCF_900475255.1	GCF_900020455.1	GCF_000290195.1
	GCF_900475725.1	GCF_900020465.1	GCF_000290215.1
	GCF_900635345.1	GCF_900020475.1	GCF_000290235.1
	GCF_900636815.1	GCF_900020485.1	GCF_000290255.1
	GCF_901542365.1	GCF_900022575.1	GCF_000290275.1
	GCF_901542425.1	GCF_900022585.1	GCF_000290295.1
	GCF_901542475.1	GCF_900022595.1	GCF_000290315.1
	GCF_901543405.1	GCF_900022605.1	GCF_000290335.1
	GCF_901543615.1	GCF_900022615.1	GCF_000290355.1
	GCF_901543655.1	GCF_900022625.1	GCF_000290375.1
	GCF_901543725.1	GCF_900022635.1	GCF_000290395.1
	GCF_901543995.1	GCF_900022645.1	GCF_000290415.1
	GCF_901544045.1	GCF_900022655.1	GCF_000290435.1
	GCF_901544215.1	GCF_900022665.1	GCF_000290455.1
	GCF_901544225.1	GCF_900022675.1	GCF_000290475.1
	GCF_901544365.1	GCF_900024965.1	GCF_000290495.1
	GCF_901875505.1	GCF_900024995.1	GCF_000290515.1
		GCF_900025005.1	GCF_000290535.1

		GCF_900025015.1	GCF_000291585.1
		GCF_900025025.1	GCF_000299135.1
		GCF_900031645.1	GCF_000310265.1
		GCF_900031655.1	GCF_000310285.1
		GCF_900031665.1	GCF_000310305.1
		GCF_900031675.1	GCF_000310325.1
		GCF_900033745.1	GCF_000310345.1
		GCF_900033755.1	GCF_000310365.1
		GCF_900033765.1	GCF_000310385.1
		GCF_900033775.1	GCF_000310405.1
		GCF_900035585.1	GCF_000310425.1
		GCF_900035595.1	GCF_000310445.1
		GCF_900035645.1	GCF_000310465.1
		GCF_900035655.1	GCF_000310485.1
		GCF_900035665.1	GCF_000310505.1
		GCF_900035675.1	GCF_000310525.1
		GCF_900035685.1	GCF_000310545.1
		GCF_900035695.1	GCF_000310565.1
		GCF_900035705.1	GCF_000310585.1
		GCF_900035715.1	GCF_000310605.1
		GCF_900035725.1	GCF_000310625.1
		GCF_900035735.1	GCF_000310645.1
		GCF_900035745.1	GCF_000310665.1
		GCF_900035755.1	GCF_000310685.1
		GCF_900035765.1	GCF_000310705.1
		GCF_900035775.1	GCF_000310725.1
		GCF_900035785.1	GCF_000310745.1
		GCF_900035795.1	GCF_000310765.1
		GCF_900035805.1	GCF_000310785.1
		GCF_900035815.1	GCF_000310805.1
		GCF_900035825.1	GCF_000310825.1
		GCF_900035835.1	GCF_000310845.1
		GCF_900035845.1	GCF_000310865.1
		GCF_900035855.1	GCF_000310885.1
		GCF_900035865.1	GCF_000310905.1
		GCF_900035875.1	GCF_000310925.1
		GCF_900035885.1	GCF_000310945.1
		GCF_900035895.1	GCF_000310965.1
		GCF_900035905.1	GCF_000310985.1

		GCF_900035915.1	GCF_000311005.1
		GCF_900035925.1	GCF_000311025.1
		GCF_900035935.1	GCF_000311045.1
		GCF_900036395.1	GCF_000311065.1
		GCF_900036405.1	GCF_000311085.1
		GCF_900036415.1	GCF_000311105.1
		GCF_900036425.1	GCF_000311125.1
		GCF_900036885.1	GCF_000311145.1
		GCF_900036895.1	GCF_000311165.1
		GCF_900036905.1	GCF_000311185.1
		GCF_900036915.1	GCF_000311205.1
		GCF_900036925.1	GCF_000311225.1
		GCF_900036935.1	GCF_000311245.1
		GCF_900036945.1	GCF_000311265.1
		GCF_900037595.1	GCF_000311305.1
		GCF_900037605.1	GCF_000311325.1
		GCF_900037635.1	GCF_000311345.1
		GCF_900037645.1	GCF_000311365.1
		GCF_900037655.1	GCF_000311385.1
		GCF_900037665.1	GCF_000311405.1
		GCF_900037675.1	GCF_000311425.1
		GCF_900037685.1	GCF_000311445.1
		GCF_900037695.1	GCF_000311465.1
		GCF_900037705.1	GCF_000311485.1
		GCF_900037715.1	GCF_000311505.1
		GCF_900037725.1	GCF_000311525.1
		GCF_900037735.1	GCF_000311545.1
		GCF_900037745.1	GCF_000311565.1
		GCF_900037755.1	GCF_000311585.1
		GCF_900037765.1	GCF_000311605.1
		GCF_900037775.1	GCF_000311625.1
		GCF_900037785.1	GCF_000311645.1
		GCF_900037795.1	GCF_000311665.1
		GCF_900037805.1	GCF_000311685.1
		GCF_900037815.1	GCF_000311705.1
		GCF_900037825.1	GCF_000322485.1
		GCF_900037835.1	GCF_000322505.1
		GCF_900037845.1	GCF_000322525.1
		GCF_900037855.1	GCF_000322545.1

		GCF_900037865.1	*GCF_000322565.1*
		GCF_900037875.1	*GCF_000322585.1*
		GCF_900037885.1	*GCF_000322605.1*
		GCF_900037895.1	*GCF_000322625.1*
		GCF_900037905.1	*GCF_000322645.1*
		GCF_900037915.1	*GCF_000322665.1*
		GCF_900037925.1	*GCF_000322685.1*
		GCF_900037935.1	*GCF_000322705.1*
		GCF_900037945.1	*GCF_000322725.1*
		GCF_900037955.1	*GCF_000322745.1*
		GCF_900037965.1	*GCF_000322805.1*
		GCF_900037975.1	*GCF_000322825.1*
		GCF_900038095.1	*GCF_000322845.1*
		GCF_900038195.1	*GCF_000322865.1*
		GCF_900038205.1	*GCF_000322885.1*
		GCF_900038215.1	*GCF_000322905.1*
		GCF_900038225.1	*GCF_000322925.1*
		GCF_900038235.1	*GCF_000322945.1*
		GCF_900038245.1	*GCF_000322965.1*
		GCF_900038255.1	*GCF_000322985.1*
		GCF_900038265.1	*GCF_000323005.1*
		GCF_900038275.1	*GCF_000323025.1*
		GCF_900038285.1	*GCF_000323045.1*
		GCF_900038295.1	*GCF_000323085.1*
		GCF_900038305.1	*GCF_000323105.1*
		GCF_900038315.1	*GCF_000323125.1*
		GCF_900038325.1	*GCF_000323145.1*
		GCF_900038335.1	*GCF_000323165.1*
		GCF_900038345.1	*GCF_000347415.1*
		GCF_900038355.1	*GCF_000347435.1*
		GCF_900038365.1	*GCF_000372605.1*
		GCF_900038375.1	*GCF_000419015.1*
		GCF_900038385.1	*GCF_000419055.2*
		GCF_900038395.1	*GCF_000419075.2*
		GCF_900038405.1	*GCF_000419095.2*
		GCF_900038415.1	*GCF_000419115.2*
		GCF_900038425.1	*GCF_000419135.2*
		GCF_900038435.1	*GCF_000419155.2*
		GCF_900038445.1	*GCF_000419175.2*

		GCF_900038455.1	*GCF_000419195.2*
		GCF_900038465.1	*GCF_000419215.1*
		GCF_900038475.1	*GCF_000419235.1*
		GCF_900038485.1	*GCF_000419255.1*
		GCF_900038495.1	*GCF_000427035.1*
		GCF_900038505.1	*GCF_000427075.1*
		GCF_900038515.1	*GCF_000454745.1*
		GCF_900038525.1	*GCF_000454765.1*
		GCF_900038535.1	*GCF_000454785.1*
		GCF_900038545.1	*GCF_000454805.1*
		GCF_900038555.1	*GCF_000454825.1*
		GCF_900038565.1	*GCF_000454845.1*
		GCF_900038575.1	*GCF_000454865.1*
		GCF_900038585.1	*GCF_000454885.1*
		GCF_900038715.1	*GCF_000454905.1*
		GCF_900038725.1	*GCF_000454925.1*
		GCF_900038735.1	*GCF_000454945.1*
		GCF_900038745.1	*GCF_000454965.1*
		GCF_900038755.1	*GCF_000454985.1*
		GCF_900038765.1	*GCF_000455005.1*
		GCF_900038775.1	*GCF_000455025.1*
		GCF_900038785.1	*GCF_000455045.1*
		GCF_900038795.1	*GCF_000455065.1*
		GCF_900038805.1	*GCF_000455085.1*
		GCF_900038815.1	*GCF_000455105.1*
		GCF_900038825.1	*GCF_000510405.1*
		GCF_900038835.1	*GCF_000689235.1*
		GCF_900038845.1	*GCF_000714695.1*
		GCF_900038855.1	*GCF_000715295.1*
		GCF_900038865.1	*GCF_000715315.1*
		GCF_900038875.1	*GCF_000730215.1*
		GCF_900038885.1	*GCF_000730255.1*
		GCF_900038895.1	*GCF_000736485.1*
		GCF_900038905.1	*GCF_000782855.1*
		GCF_900038915.1	*GCF_000825945.1*
		GCF_900038925.1	*GCF_000825965.1*
		GCF_900038935.1	*GCF_000825985.1*
		GCF_900038945.1	*GCF_000826005.1*
		GCF_900038955.1	*GCF_000826025.1*

		GCF_900038965.1	*GCF_000831105.1*
		GCF_900038975.1	*GCF_000831125.1*
		GCF_900038985.1	*GCF_000831145.1*
		GCF_900038995.1	*GCF_001010325.1*
		GCF_900039005.1	*GCF_001010335.1*
		GCF_900039015.1	*GCF_001010345.1*
		GCF_900039025.1	*GCF_001015415.1*
		GCF_900039035.1	*GCF_001015425.1*
		GCF_900039045.1	*GCF_001015435.1*
		GCF_900039055.1	*GCF_001015445.1*
		GCF_900039065.1	*GCF_001015495.1*
		GCF_900039075.1	*GCF_001015505.1*
		GCF_900039085.1	*GCF_001015525.1*
		GCF_900039095.1	*GCF_001015555.1*
		GCF_900039105.1	*GCF_001015575.1*
		GCF_900039115.1	*GCF_001015595.1*
		GCF_900039125.1	*GCF_001015605.1*
		GCF_900039135.1	*GCF_001015615.1*
		GCF_900039145.1	*GCF_001015655.1*
		GCF_900039155.1	*GCF_001015675.1*
		GCF_900039165.1	*GCF_001015685.1*
		GCF_900039175.1	*GCF_001015695.1*
		GCF_900039185.1	*GCF_001015735.1*
		GCF_900039195.1	*GCF_001015755.1*
		GCF_900039205.1	*GCF_001015765.1*
		GCF_900039215.1	*GCF_001015775.1*
		GCF_900039665.1	*GCF_001015815.1*
		GCF_900039675.1	*GCF_001015835.1*
		GCF_900039685.1	*GCF_001015845.1*
		GCF_900039695.1	*GCF_001015875.1*
		GCF_900039705.1	*GCF_001015895.1*
		GCF_900039715.1	*GCF_001015915.1*
		GCF_900039725.1	*GCF_001015925.1*
		GCF_900039735.1	*GCF_001015955.1*
		GCF_900039745.1	*GCF_001015965.1*
		GCF_900039755.1	*GCF_001015995.1*
		GCF_900039765.1	*GCF_001016005.1*
		GCF_900039775.1	*GCF_001016035.1*
		GCF_900039785.1	*GCF_001016045.1*

		GCF_900039795.1	*GCF_001016065.1*
		GCF_900039805.1	*GCF_001016095.1*
		GCF_900039815.1	*GCF_001016115.1*
		GCF_900039825.1	*GCF_001016125.1*
		GCF_900039835.1	*GCF_001016135.1*
		GCF_900039845.1	*GCF_001016175.1*
		GCF_900039855.1	*GCF_001016195.1*
		GCF_900039865.1	*GCF_001016205.1*
		GCF_900039875.1	*GCF_001016215.1*
		GCF_900039885.1	*GCF_001016255.1*
		GCF_900039895.1	*GCF_001016265.1*
		GCF_900039905.1	*GCF_001016285.1*
		GCF_900039915.1	*GCF_001016295.1*
		GCF_900039925.1	*GCF_001016335.1*
		GCF_900039935.1	*GCF_001016355.1*
		GCF_900039945.1	*GCF_001016375.1*
		GCF_900039955.1	*GCF_001016385.1*
		GCF_900039965.1	*GCF_001016415.1*
		GCF_900040055.1	*GCF_001016435.1*
		GCF_900040075.1	*GCF_001016445.1*
		GCF_900040085.1	*GCF_001016465.1*
		GCF_900040095.1	*GCF_001016495.1*
		GCF_900040105.1	*GCF_001016515.1*
		GCF_900040115.1	*GCF_001016525.1*
		GCF_900040125.1	*GCF_001016545.1*
		GCF_900040135.1	*GCF_001016575.1*
		GCF_900040145.1	*GCF_001016595.1*
		GCF_900040155.1	*GCF_001016615.1*
		GCF_900040165.1	*GCF_001016625.1*
		GCF_900040175.1	*GCF_001016655.1*
		GCF_900040185.1	*GCF_001016675.1*
		GCF_900040195.1	*GCF_001016695.1*
		GCF_900040205.1	*GCF_001016715.1*
		GCF_900040215.1	*GCF_001016725.1*
		GCF_900040225.1	*GCF_001016755.1*
		GCF_900040235.1	*GCF_001016775.1*
		GCF_900040245.1	*GCF_001016795.1*
		GCF_900040255.1	*GCF_001016815.1*
		GCF_900040265.1	*GCF_001016835.1*

		GCF_900040275.1	GCF_001016855.1
		GCF_900040285.1	GCF_001016875.1
		GCF_900040295.1	GCF_001016895.1
		GCF_900040305.1	GCF_001016915.1
		GCF_900040315.1	GCF_001016935.1
		GCF_900040325.1	GCF_001016955.1
		GCF_900040335.1	GCF_001016965.1
		GCF_900040345.1	GCF_001016995.1
		GCF_900040355.1	GCF_001017015.1
		GCF_900040365.1	GCF_001017035.1
		GCF_900040375.1	GCF_001017045.1
		GCF_900040385.1	GCF_001017075.1
		GCF_900040395.1	GCF_001017085.1
		GCF_900040405.1	GCF_001017115.1
		GCF_900040415.1	GCF_001017835.1
		GCF_900040425.1	GCF_001017845.1
		GCF_900040435.1	GCF_001017855.1
		GCF_900040445.1	GCF_001017925.1
		GCF_900040455.1	GCF_001017935.1
		GCF_900040465.1	GCF_001017975.1
		GCF_900040475.1	GCF_001017995.1
		GCF_900040485.1	GCF_001018005.1
		GCF_900040495.1	GCF_001018015.1
		GCF_900040505.1	GCF_001018055.1
		GCF_900040515.1	GCF_001018065.1
		GCF_900040525.1	GCF_001018085.1
		GCF_900040535.1	GCF_001018095.1
		GCF_900040545.1	GCF_001018135.1
		GCF_900040555.1	GCF_001018155.1
		GCF_900040565.1	GCF_001018165.1
		GCF_900040575.1	GCF_001018175.1
		GCF_900040585.1	GCF_001018215.1
		GCF_900040595.1	GCF_001018225.1
		GCF_900040605.1	GCF_001018245.1
		GCF_900040695.1	GCF_001018255.1
		GCF_900040725.1	GCF_001018295.1
		GCF_900040735.1	GCF_001018315.1
		GCF_900040745.1	GCF_001018325.1
		GCF_900040755.1	GCF_001018335.1

		GCF_900040765.1	*GCF_001018375.1*
		GCF_900040775.1	*GCF_001018395.1*
		GCF_900040785.1	*GCF_001018405.1*
		GCF_900040795.1	*GCF_001018415.1*
		GCF_900040805.1	*GCF_001018455.1*
		GCF_900040815.1	*GCF_001018475.1*
		GCF_900040825.1	*GCF_001018485.1*
		GCF_900040835.1	*GCF_001018495.1*
		GCF_900040845.1	*GCF_001018535.1*
		GCF_900040855.1	*GCF_001018555.1*
		GCF_900040865.1	*GCF_001018565.1*
		GCF_900040875.1	*GCF_001018575.1*
		GCF_900040885.1	*GCF_001018615.1*
		GCF_900040895.1	*GCF_001026925.1*
		GCF_900040905.1	*GCF_001070935.1*
		GCF_900040915.1	*GCF_001072615.1*
		GCF_900040925.1	*GCF_001072815.1*
		GCF_900040935.1	*GCF_001074175.1*
		GCF_900040945.1	*GCF_001074215.1*
		GCF_900040955.1	*GCF_001074255.1*
		GCF_900040965.1	*GCF_001074285.1*
		GCF_900040975.1	*GCF_001074585.1*
		GCF_900040985.1	*GCF_001076065.1*
		GCF_900040995.1	*GCF_001086185.1*
		GCF_900041005.1	*GCF_001087245.1*
		GCF_900041015.1	*GCF_001088005.1*
		GCF_900041025.1	*GCF_001088105.1*
		GCF_900041035.1	*GCF_001089905.1*
		GCF_900041045.1	*GCF_001091485.1*
		GCF_900041055.1	*GCF_001093745.1*
		GCF_900041065.1	*GCF_001097365.1*
		GCF_900041075.1	*GCF_001098005.1*
		GCF_900041085.1	*GCF_001098165.1*
		GCF_900041095.1	*GCF_001099185.1*
		GCF_900041105.1	*GCF_001100165.1*
		GCF_900041115.1	*GCF_001101765.1*
		GCF_900041125.1	*GCF_001102725.1*
		GCF_900041135.1	*GCF_001102825.1*
		GCF_900041145.1	*GCF_001103585.1*

		GCF_900041155.1	*GCF_001103625.1*
		GCF_900041165.1	*GCF_001104145.1*
		GCF_900041175.1	*GCF_001104505.1*
		GCF_900041185.1	*GCF_001106525.1*
		GCF_900041195.1	*GCF_001108625.1*
		GCF_900041205.1	*GCF_001108745.1*
		GCF_900041215.1	*GCF_001109505.1*
		GCF_900041225.1	*GCF_001109825.1*
		GCF_900041235.1	*GCF_001110105.1*
		GCF_900041775.1	*GCF_001110405.1*
		GCF_900041785.1	*GCF_001111745.1*
		GCF_900041795.1	*GCF_001114925.1*
		GCF_900041805.1	*GCF_001115485.1*
		GCF_900041815.1	*GCF_001115545.1*
		GCF_900041825.1	*GCF_001116525.1*
		GCF_900041835.1	*GCF_001118625.1*
		GCF_900041845.1	*GCF_001119085.1*
		GCF_900041855.1	*GCF_001119265.1*
		GCF_900041865.1	*GCF_001123205.1*
		GCF_900041875.1	*GCF_001123605.1*
		GCF_900041885.1	*GCF_001123965.1*
		GCF_900041895.1	*GCF_001124065.1*
		GCF_900041905.1	*GCF_001124925.1*
		GCF_900041915.1	*GCF_001127125.1*
		GCF_900041925.1	*GCF_001127665.1*
		GCF_900041935.1	*GCF_001130545.1*
		GCF_900041945.1	*GCF_001133525.1*
		GCF_900041955.1	*GCF_001133625.1*
		GCF_900041965.1	*GCF_001134425.1*
		GCF_900041975.1	*GCF_001134625.1*
		GCF_900041985.1	*GCF_001135525.1*
		GCF_900041995.1	*GCF_001138645.1*
		GCF_900042005.1	*GCF_001140025.1*
		GCF_900042015.1	*GCF_001140565.1*
		GCF_900042025.1	*GCF_001142125.1*
		GCF_900042035.1	*GCF_001142165.1*
		GCF_900042045.1	*GCF_001145265.1*
		GCF_900042055.1	*GCF_001145805.1*
		GCF_900042065.1	*GCF_001146325.1*

		GCF_900042075.1	*GCF_001146845.1*
		GCF_900042085.1	*GCF_001147165.1*
		GCF_900042095.1	*GCF_001147785.1*
		GCF_900042105.1	*GCF_001149385.1*
		GCF_900042115.1	*GCF_001151005.1*
		GCF_900042125.1	*GCF_001152205.1*
		GCF_900042135.1	*GCF_001153505.1*
		GCF_900042145.1	*GCF_001155125.1*
		GCF_900042155.1	*GCF_001155785.1*
		GCF_900042165.1	*GCF_001155905.1*
		GCF_900042175.1	*GCF_001156365.1*
		GCF_900042185.1	*GCF_001156965.1*
		GCF_900042195.1	*GCF_001159365.1*
		GCF_900042205.1	*GCF_001160905.1*
		GCF_900042215.1	*GCF_001162145.1*
		GCF_900042225.1	*GCF_001162445.1*
		GCF_900042235.1	*GCF_001163105.1*
		GCF_900042245.1	*GCF_001164665.1*
		GCF_900042255.1	*GCF_001166505.1*
		GCF_900042265.1	*GCF_001166605.1*
		GCF_900042275.1	*GCF_001171645.1*
		GCF_900042285.1	*GCF_001173205.1*
		GCF_900042295.1	*GCF_001174785.1*
		GCF_900042305.1	*GCF_001190805.1*
		GCF_900042315.1	*GCF_001190825.1*
		GCF_900042475.1	*GCF_001190845.1*
		GCF_900042485.1	*GCF_001190885.1*
		GCF_900042495.1	*GCF_001217725.1*
		GCF_900042505.1	*GCF_001218205.1*
		GCF_900042515.1	*GCF_001218945.1*
		GCF_900042525.1	*GCF_001221465.1*
		GCF_900042535.1	*GCF_001221525.1*
		GCF_900042545.1	*GCF_001221825.1*
		GCF_900042555.1	*GCF_001221965.1*
		GCF_900042565.1	*GCF_001222045.1*
		GCF_900042575.1	*GCF_001222545.1*
		GCF_900042585.1	*GCF_001222685.1*
		GCF_900042595.1	*GCF_001223485.1*
		GCF_900042605.1	*GCF_001266635.1*

		GCF_900042615.1	*GCF_001275545.2*
		GCF_900042625.1	*GCF_001348535.1*
		GCF_900042635.1	*GCF_001348555.1*
		GCF_900042645.1	*GCF_001348575.1*
		GCF_900042655.1	*GCF_001348595.1*
		GCF_900042665.1	*GCF_001348615.1*
		GCF_900042675.1	*GCF_001348635.1*
		GCF_900042685.1	*GCF_001348655.1*
		GCF_900042695.1	*GCF_001348675.1*
		GCF_900042705.1	*GCF_001348695.1*
		GCF_900042715.1	*GCF_001348715.1*
		GCF_900042725.1	*GCF_001348735.1*
		GCF_900042735.1	*GCF_001348755.1*
		GCF_900043545.1	*GCF_001348775.1*
		GCF_900043555.1	*GCF_001348795.1*
		GCF_900043565.1	*GCF_001348815.1*
		GCF_900043575.1	*GCF_001348835.1*
		GCF_900043585.1	*GCF_001348855.1*
		GCF_900043595.1	*GCF_001348875.1*
		GCF_900043605.1	*GCF_001348895.1*
		GCF_900043615.1	*GCF_001348915.1*
		GCF_900043625.1	*GCF_001348935.1*
		GCF_900043635.1	*GCF_001348955.1*
		GCF_900043645.1	*GCF_001348975.1*
		GCF_900043655.1	*GCF_001348995.1*
		GCF_900043885.1	*GCF_001349015.1*
		GCF_900043895.1	*GCF_001349035.1*
		GCF_900043905.1	*GCF_001349055.1*
		GCF_900043915.1	*GCF_001398435.1*
		GCF_900043925.1	*GCF_001398455.1*
		GCF_900043935.1	*GCF_001398475.1*
		GCF_900043945.1	*GCF_001398495.1*
		GCF_900043955.1	*GCF_001398515.1*
		GCF_900044095.1	*GCF_001398535.1*
		GCF_900044325.1	*GCF_001398555.1*
		GCF_900044345.1	*GCF_001398575.1*
		GCF_900044355.1	*GCF_001398595.1*
		GCF_900044365.1	*GCF_001398615.1*
		GCF_900044375.1	*GCF_001398635.1*

		GCF_900044385.1	GCF_001398655.1
		GCF_900044395.1	GCF_001398675.1
		GCF_900044405.1	GCF_001398695.1
		GCF_900044415.1	GCF_001398715.1
		GCF_900044425.1	GCF_001398735.1
		GCF_900044435.1	GCF_001398755.1
		GCF_900044445.1	GCF_001398775.1
		GCF_900044455.1	GCF_001398795.1
		GCF_900044465.1	GCF_001398815.1
		GCF_900044475.1	GCF_001398835.1
		GCF_900044485.1	GCF_001398855.1
		GCF_900044495.1	GCF_001398875.1
		GCF_900044505.1	GCF_001398895.1
		GCF_900044515.1	GCF_001398915.1
		GCF_900044525.1	GCF_001398935.1
		GCF_900044535.1	GCF_001398955.1
		GCF_900044545.1	GCF_001398975.1
		GCF_900044555.1	GCF_001398995.1
		GCF_900044565.1	GCF_001399015.1
		GCF_900044575.1	GCF_001399035.1
		GCF_900044595.1	GCF_001399055.1
		GCF_900044605.1	GCF_001399075.1
		GCF_900044615.1	GCF_001399095.1
		GCF_900044625.1	GCF_001399115.1
		GCF_900044635.1	GCF_001399135.1
		GCF_900044775.1	GCF_001399155.1
		GCF_900044785.1	GCF_001399175.1
		GCF_900044795.1	GCF_001399195.1
		GCF_900044885.1	GCF_001399295.1
		GCF_900044895.1	GCF_001399315.1
		GCF_900044905.1	GCF_001399335.1
		GCF_900044915.1	GCF_001399355.1
		GCF_900044925.1	GCF_001399375.1
		GCF_900044935.1	GCF_001399395.1
		GCF_900044945.1	GCF_001399415.1
		GCF_900044955.1	GCF_001399435.1
		GCF_900044965.1	GCF_001439905.1
		GCF_900044975.1	GCF_001448985.1
		GCF_900044985.1	GCF_001484865.1

		GCF_900044995.1	GCF_001546675.1
		GCF_900045005.1	GCF_001546685.1
		GCF_900045015.1	GCF_001546695.1
		GCF_900045025.1	GCF_001546715.1
		GCF_900045035.1	GCF_001546755.1
		GCF_900045045.1	GCF_001546775.1
		GCF_900045055.1	GCF_001546785.1
		GCF_900045065.1	GCF_001552035.1
		GCF_900045075.1	GCF_001592385.1
		GCF_900045085.1	GCF_001592425.1
		GCF_900045095.1	GCF_001592615.1
		GCF_900045105.1	GCF_001592635.1
		GCF_900045115.1	GCF_001675305.1
		GCF_900045125.1	GCF_001683515.1
		GCF_900045135.1	GCF_001692935.1
		GCF_900045145.1	GCF_001693835.1
		GCF_900045155.1	GCF_001693845.1
		GCF_900045165.1	GCF_001693855.1
		GCF_900045175.1	GCF_001693865.1
		GCF_900045185.1	GCF_001693915.1
		GCF_900045195.1	GCF_001693925.1
		GCF_900045205.1	GCF_001693955.1
		GCF_900045215.1	GCF_001693965.1
		GCF_900045225.1	GCF_001693995.1
		GCF_900045245.1	GCF_001694015.1
		GCF_900045255.1	GCF_001694025.1
		GCF_900045265.1	GCF_001694045.1
		GCF_900045275.1	GCF_001694075.1
		GCF_900045475.1	GCF_001694095.1
		GCF_900045495.1	GCF_001694115.1
		GCF_900045505.1	GCF_001694125.1
		GCF_900045515.1	GCF_001694155.1
		GCF_900045525.1	GCF_001694175.1
		GCF_900045535.1	GCF_001694195.1
		GCF_900045545.1	GCF_001694205.1
		GCF_900045555.1	GCF_001694235.1
		GCF_900045565.1	GCF_001694255.1
		GCF_900045575.1	GCF_001694265.1
		GCF_900045585.1	GCF_001694285.1

		GCF_900045595.1	*GCF_001694315.1*
		GCF_900045605.1	*GCF_001694325.1*
		GCF_900045615.1	*GCF_001694355.1*
		GCF_900045625.1	*GCF_001694365.1*
		GCF_900045635.1	*GCF_001694395.1*
		GCF_900045645.1	*GCF_001694425.1*
		GCF_900045655.1	*GCF_001694435.1*
		GCF_900045665.1	*GCF_001694475.1*
		GCF_900045675.1	*GCF_001694495.1*
		GCF_900045685.1	*GCF_001694515.1*
		GCF_900045695.1	*GCF_001694525.1*
		GCF_900045705.1	*GCF_001694555.1*
		GCF_900045715.1	*GCF_001694575.1*
		GCF_900045725.1	*GCF_001694585.1*
		GCF_900045735.1	*GCF_001694625.1*
		GCF_900045745.1	*GCF_001694645.1*
		GCF_900045755.1	*GCF_001694675.1*
		GCF_900045765.1	*GCF_001694695.1*
		GCF_900045775.1	*GCF_001694705.1*
		GCF_900045785.1	*GCF_001694725.1*
		GCF_900045795.1	*GCF_001694745.1*
		GCF_900045805.1	*GCF_001694775.1*
		GCF_900045815.1	*GCF_001694785.1*
		GCF_900045825.1	*GCF_001694815.1*
		GCF_900045935.1	*GCF_001694825.1*
		GCF_900045945.1	*GCF_001694855.1*
		GCF_900045955.1	*GCF_001694875.1*
		GCF_900045965.1	*GCF_001694895.1*
		GCF_900045975.1	*GCF_001694905.1*
		GCF_900045985.1	*GCF_001694935.1*
		GCF_900045995.1	*GCF_001694945.1*
		GCF_900046005.1	*GCF_001694975.1*
		GCF_900046015.1	*GCF_001694985.1*
		GCF_900046025.1	*GCF_001695015.1*
		GCF_900046035.1	*GCF_001695025.1*
		GCF_900046045.1	*GCF_001695055.1*
		GCF_900046055.1	*GCF_001695075.1*
		GCF_900046065.1	*GCF_001695095.1*
		GCF_900046075.1	*GCF_001695105.1*

		GCF_900046085.1	*GCF_001695135.1*
		GCF_900046095.1	*GCF_001695155.1*
		GCF_900046105.1	*GCF_001695175.1*
		GCF_900046115.1	*GCF_001695185.1*
		GCF_900046125.1	*GCF_001708205.1*
		GCF_900046135.1	*GCF_001712835.1*
		GCF_900046145.1	*GCF_001729925.2*
		GCF_900046155.1	*GCF_001856375.1*
		GCF_900046165.1	*GCF_001856395.1*
		GCF_900046175.1	*GCF_001856425.1*
		GCF_900046185.1	*GCF_001856445.1*
		GCF_900046195.1	*GCF_001856455.1*
		GCF_900046205.1	*GCF_001856475.1*
		GCF_900046215.1	*GCF_001856505.1*
		GCF_900046225.1	*GCF_001880505.1*
		GCF_900046235.1	*GCF_001880515.1*
		GCF_900046245.1	*GCF_001880525.1*
		GCF_900046255.1	*GCF_001880535.1*
		GCF_900046265.1	*GCF_001880585.1*
		GCF_900046275.1	*GCF_001880605.1*
		GCF_900046285.1	*GCF_001880615.1*
		GCF_900046295.1	*GCF_001880625.1*
		GCF_900046305.1	*GCF_001880665.1*
		GCF_900046315.1	*GCF_001880675.1*
		GCF_900046325.1	*GCF_001880685.1*
		GCF_900046335.1	*GCF_001880725.1*
		GCF_900046345.1	*GCF_001880745.1*
		GCF_900046355.1	*GCF_001880765.1*
		GCF_900046365.1	*GCF_001932715.1*
		GCF_900046375.1	*GCF_002025005.1*
		GCF_900046385.1	*GCF_002025025.1*
		GCF_900046395.1	*GCF_002073695.2*
		GCF_900046405.1	*GCF_002104835.1*
		GCF_900046415.1	*GCF_002135445.1*
		GCF_900046425.1	*GCF_002135505.1*
		GCF_900046465.1	*GCF_002176575.1*
		GCF_900046795.1	*GCF_002176585.1*
		GCF_900046835.1	*GCF_002176615.1*
		GCF_900046845.1	*GCF_002197205.1*

		GCF_900046855.1	*GCF_002197245.1*
		GCF_900046865.1	*GCF_002197265.1*
		GCF_900046875.1	*GCF_002197285.1*
		GCF_900046885.1	*GCF_002197305.1*
		GCF_900047135.1	*GCF_002197325.1*
		GCF_900047155.1	*GCF_002197365.1*
		GCF_900047165.1	*GCF_002197385.1*
		GCF_900047175.1	*GCF_002197425.1*
		GCF_900047185.1	*GCF_002214425.1*
		GCF_900047195.1	*GCF_002239135.1*
		GCF_900047205.1	*GCF_002239145.1*
		GCF_900047215.1	*GCF_002239175.1*
		GCF_900047225.1	*GCF_002239185.1*
		GCF_900047235.1	*GCF_002239205.1*
		GCF_900047245.1	*GCF_002239235.1*
		GCF_900047255.1	*GCF_002239245.1*
		GCF_900047265.1	*GCF_002239265.1*
		GCF_900047275.1	*GCF_002239285.1*
		GCF_900047285.1	*GCF_002239305.1*
		GCF_900047295.1	*GCF_002239325.1*
		GCF_900047305.1	*GCF_002239345.1*
		GCF_900047315.1	*GCF_002239365.1*
		GCF_900047325.1	*GCF_002239385.1*
		GCF_900047335.1	*GCF_002246945.1*
		GCF_900047345.1	*GCF_002266915.1*
		GCF_900047355.1	*GCF_002266925.1*
		GCF_900047365.1	*GCF_002266935.1*
		GCF_900047375.1	*GCF_002278655.1*
		GCF_900047385.1	*GCF_002278755.1*
		GCF_900047395.1	*GCF_002278775.1*
		GCF_900047405.1	*GCF_002278795.1*
		GCF_900047415.1	*GCF_002289205.1*
		GCF_900047425.1	*GCF_002812425.1*
		GCF_900047435.1	*GCF_002812445.1*
		GCF_900047445.1	*GCF_002812465.1*
		GCF_900047455.1	*GCF_002812505.1*
		GCF_900047465.1	*GCF_002861005.1*
		GCF_900047475.1	*GCF_002871335.1*
		GCF_900047485.1	*GCF_002871535.1*

		GCF_900047495.1	*GCF_002884585.1*
		GCF_900047505.1	*GCF_002930675.1*
		GCF_900047515.1	*GCF_002930685.1*
		GCF_900048085.1	*GCF_002930695.1*
		GCF_900048095.1	*GCF_002937095.1*
		GCF_900048135.1	*GCF_003160735.1*
		GCF_900048145.1	*GCF_003160745.1*
		GCF_900048155.1	*GCF_003174175.1*
		GCF_900048165.1	*GCF_003174215.1*
		GCF_900048175.1	*GCF_003174235.1*
		GCF_900048185.1	*GCF_003174655.1*
		GCF_900048195.1	*GCF_003174715.1*
		GCF_900048205.1	*GCF_003174735.1*
		GCF_900048215.1	*GCF_003186745.1*
		GCF_900048225.1	*GCF_003284605.1*
		GCF_900048235.1	*GCF_003287995.1*
		GCF_900048245.1	*GCF_003288015.1*
		GCF_900048255.1	*GCF_003288035.1*
		GCF_900048265.1	*GCF_003288055.1*
		GCF_900048275.1	*GCF_003288075.1*
		GCF_900048285.1	*GCF_003319215.1*
		GCF_900048295.1	*GCF_003382425.1*
		GCF_900048305.1	*GCF_003382435.1*
		GCF_900048315.1	*GCF_003382445.1*
		GCF_900048325.1	*GCF_003403115.1*
		GCF_900048335.1	*GCF_003605605.1*
		GCF_900048345.1	*GCF_003640705.1*
		GCF_900048355.1	*GCF_003640715.1*
		GCF_900048365.1	*GCF_003640725.1*
		GCF_900048375.1	*GCF_003640785.1*
		GCF_900048385.1	*GCF_003725835.1*
		GCF_900048395.1	*GCF_003812805.1*
		GCF_900048405.1	*GCF_003859425.1*
		GCF_900048445.1	*GCF_003859445.1*
		GCF_900048455.1	*GCF_003859455.1*
		GCF_900048465.1	*GCF_003859485.1*
		GCF_900048475.1	*GCF_003859505.1*
		GCF_900048485.1	*GCF_003859515.1*
		GCF_900048495.1	*GCF_003859535.1*

		GCF_900048505.1	*GCF_003859555.1*
		GCF_900048515.1	*GCF_003859585.1*
		GCF_900048525.1	*GCF_003859595.1*
		GCF_900048535.1	*GCF_003859625.1*
		GCF_900048545.1	*GCF_003859635.1*
		GCF_900048555.1	*GCF_003859655.1*
		GCF_900048565.1	*GCF_003859675.1*
		GCF_900048575.1	*GCF_003859695.1*
		GCF_900048585.1	*GCF_003859725.1*
		GCF_900048595.1	*GCF_003931905.1*
		GCF_900048605.1	*GCF_003931955.1*
		GCF_900048615.1	*GCF_003939065.1*
		GCF_900048625.1	*GCF_003966545.1*
		GCF_900048635.1	*GCF_003992515.1*
		GCF_900048645.1	*GCF_003992555.1*
		GCF_900048655.1	*GCF_004120375.1*
		GCF_900048665.1	*GCF_004120385.1*
		GCF_900048675.1	*GCF_004120395.1*
		GCF_900048685.1	*GCF_004120405.1*
		GCF_900048695.1	*GCF_004120435.1*
		GCF_900048705.1	*GCF_004349445.1*
		GCF_900048715.1	*GCF_004349485.1*
		GCF_900048725.1	*GCF_004349495.1*
		GCF_900048735.1	*GCF_004349525.1*
		GCF_900048745.1	*GCF_004349535.1*
		GCF_900048755.1	*GCF_004353995.1*
		GCF_900048765.1	*GCF_004369755.1*
		GCF_900048775.1	*GCF_004369765.1*
		GCF_900048785.1	*GCF_004369775.1*
		GCF_900048975.1	*GCF_004369785.1*
		GCF_900049315.1	*GCF_004369845.1*
		GCF_900049325.1	*GCF_004369855.1*
		GCF_900049385.1	*GCF_004369875.1*
		GCF_900049395.1	*GCF_005233865.1*
		GCF_900049405.1	*GCF_005233935.1*
		GCF_900049415.1	*GCF_005233945.1*
		GCF_900049425.1	*GCF_005233955.1*
		GCF_900049435.1	*GCF_006543105.1*
		GCF_900049445.1	*GCF_006543115.1*

		GCF_900049455.1	*GCF_006543125.1*
		GCF_900049465.1	*GCF_006543135.1*
		GCF_900049765.1	*GCF_006543145.1*
		GCF_900049775.1	*GCF_006543205.1*
		GCF_900049785.1	*GCF_006543215.1*
		GCF_900049795.1	*GCF_006543245.1*
		GCF_900049805.1	*GCF_006543265.1*
		GCF_900049815.1	*GCF_006543275.1*
		GCF_900050255.1	*GCF_006543305.1*
		GCF_900050265.1	*GCF_006543325.1*
		GCF_900050275.1	*GCF_006543335.1*
		GCF_900050285.1	*GCF_006543345.1*
		GCF_900050575.1	*GCF_006543375.1*
		GCF_900050585.1	*GCF_006543405.1*
		GCF_900050595.1	*GCF_006543425.1*
		GCF_900050605.1	*GCF_006543435.1*
		GCF_900050615.1	*GCF_006543455.1*
		GCF_900050625.1	*GCF_006543465.1*
		GCF_900050635.1	*GCF_006543505.1*
		GCF_900050645.1	*GCF_006543525.1*
		GCF_900050655.1	*GCF_006543535.1*
		GCF_900051035.1	*GCF_006543545.1*
		GCF_900051045.1	*GCF_006543565.1*
		GCF_900051055.1	*GCF_006716245.1*
		GCF_900051065.1	*GCF_006874565.1*
		GCF_900051075.1	*GCF_007050265.1*
		GCF_900051085.1	*GCF_007050295.1*
		GCF_900051095.1	*GCF_008086005.1*
		GCF_900051345.1	*GCF_008086035.1*
		GCF_900051355.1	*GCF_008086085.1*
		GCF_900051365.1	*GCF_008086095.1*
		GCF_900051375.1	*GCF_008692275.1*
		GCF_900051385.1	*GCF_008693505.1*
		GCF_900051765.1	*GCF_008693585.1*
		GCF_900051775.1	*GCF_008709735.1*
		GCF_900051785.1	*GCF_008709755.1*
		GCF_900051795.1	*GCF_008709775.1*
		GCF_900051805.1	*GCF_008709805.1*
		GCF_900051815.1	*GCF_008709835.1*

		GCF_900052115.1	*GCF_008709845.1*
		GCF_900052125.1	*GCF_008709865.1*
		GCF_900052345.1	*GCF_008709885.1*
		GCF_900052355.1	*GCF_008709935.1*
		GCF_900052365.1	*GCF_008709945.1*
		GCF_900052375.1	*GCF_008709955.1*
		GCF_900052635.1	*GCF_008709965.1*
		GCF_900052645.1	*GCF_008709995.1*
		GCF_900052655.1	*GCF_008710045.1*
		GCF_900052665.1	*GCF_008710055.1*
		GCF_900052675.1	*GCF_008710065.1*
		GCF_900052955.1	*GCF_008710255.1*
		GCF_900053455.1	*GCF_008710265.1*
		GCF_900053465.1	*GCF_008710335.1*
		GCF_900053475.1	*GCF_008710345.1*
		GCF_900053485.1	*GCF_008710375.1*
		GCF_900053925.1	*GCF_008710385.1*
		GCF_900053935.1	*GCF_008710395.1*
		GCF_900054175.1	*GCF_008710435.1*
		GCF_900054445.1	*GCF_008710445.1*
		GCF_900054455.1	*GCF_008710475.1*
		GCF_900054465.1	*GCF_008710485.1*
		GCF_900054475.1	*GCF_008710505.1*
		GCF_900054485.1	*GCF_008710515.1*
		GCF_900054495.1	*GCF_008710545.1*
		GCF_900054705.1	*GCF_008710575.1*
		GCF_900054715.1	*GCF_008710585.1*
		GCF_900054915.1	*GCF_008710595.1*
		GCF_900054925.1	*GCF_008710635.1*
		GCF_900054935.1	*GCF_008710645.1*
		GCF_900054945.1	*GCF_008710675.1*
		GCF_900055265.1	*GCF_008710685.1*
		GCF_900055275.1	*GCF_008710735.1*
		GCF_900055285.1	*GCF_008710755.1*
		GCF_900055295.1	*GCF_008710795.1*
		GCF_900055305.1	*GCF_008710805.1*
		GCF_900055645.1	*GCF_008710835.1*
		GCF_900055945.1	*GCF_008710845.1*
		GCF_900055955.1	*GCF_008710865.1*

		GCF_900056255.1	*GCF_008710895.1*
		GCF_900056265.1	*GCF_008710905.1*
		GCF_900056275.1	*GCF_008710915.1*
		GCF_900056285.1	*GCF_008710935.1*
		GCF_900056295.1	*GCF_008710965.1*
		GCF_900056305.1	*GCF_008710995.1*
		GCF_900056315.1	*GCF_008711005.1*
		GCF_900056665.1	*GCF_008711015.1*
		GCF_900056675.1	*GCF_008711025.1*
		GCF_900056685.1	*GCF_008711075.1*
		GCF_900056955.1	*GCF_008711085.1*
		GCF_900056965.1	*GCF_008711115.1*
		GCF_900056975.1	*GCF_008806305.1*
		GCF_900057105.1	*GCF_009495845.1*
		GCF_900057285.1	*GCF_009495855.1*
		GCF_900057295.1	*GCF_009495905.1*
		GCF_900057305.1	*GCF_009496495.1*
		GCF_900057315.1	*GCF_009496605.1*
		GCF_900057325.1	*GCF_009496625.1*
		GCF_900057335.1	*GCF_009496655.1*
		GCF_900057345.1	*GCF_009496665.1*
		GCF_900057635.1	*GCF_009496695.1*
		GCF_900057645.1	*GCF_009496705.1*
		GCF_900057795.1	*GCF_009771215.1*
		GCF_900057805.1	*GCF_009771235.1*
		GCF_900057815.1	*GCF_009771255.1*
		GCF_900058105.1	*GCF_009771285.1*
		GCF_900058115.1	*GCF_009771295.1*
		GCF_900058365.1	*GCF_009771325.1*
		GCF_900058375.1	*GCF_009771345.1*
		GCF_900058385.1	*GCF_009771375.1*
		GCF_900058615.1	*GCF_009771385.1*
		GCF_900058625.1	*GCF_009771775.1*
		GCF_900058635.1	*GCF_009771785.1*
		GCF_900058875.1	*GCF_009771795.1*
		GCF_900058885.1	*GCF_009771805.1*
		GCF_900058895.1	*GCF_009771825.1*
		GCF_900058905.1	*GCF_009771845.1*
		GCF_900058915.1	*GCF_009771865.1*

		GCF_900059245.1	*GCF_009771885.1*
		GCF_900059255.1	*GCF_009771895.1*
		GCF_900059265.1	*GCF_009771925.1*
		GCF_900059555.1	*GCF_009771945.1*
		GCF_900059615.1	*GCF_009771985.1*
		GCF_900059805.1	*GCF_009866845.1*
		GCF_900059815.1	*GCF_009930895.1*
		GCF_900060035.1	*GCF_009930915.1*
		GCF_900060045.1	*GCF_009933295.1*
		GCF_900060215.1	*GCF_009933315.1*
		GCF_900060225.1	*GCF_009933365.1*
		GCF_900060465.1	*GCF_009933395.1*
		GCF_900060475.1	*GCF_009933415.1*
		GCF_900060485.1	*GCF_009933425.1*
		GCF_900060495.1	*GCF_009933455.1*
		GCF_900060805.1	*GCF_009933465.1*
		GCF_900061095.1	*GCF_009933485.1*
		GCF_900061105.1	*GCF_009933515.1*
		GCF_900061115.1	*GCF_009933555.1*
		GCF_900061125.1	*GCF_009933575.1*
		GCF_900061735.1	*GCF_009933615.1*
		GCF_900061985.1	*GCF_009933635.1*
		GCF_900062125.1	*GCF_009933645.1*
		GCF_900062135.1	*GCF_009933665.1*
		GCF_900062145.1	*GCF_009933695.1*
		GCF_900062765.1	*GCF_009933705.1*
		GCF_900062775.1	*GCF_009933735.1*
		GCF_900062785.1	*GCF_009933745.1*
		GCF_900062795.1	*GCF_009933775.1*
		GCF_900062805.1	*GCF_009933795.1*
		GCF_900062885.1	*GCF_009933805.1*
		GCF_900062895.1	*GCF_009933835.1*
		GCF_900062905.1	*GCF_009933845.1*
		GCF_900063305.1	*GCF_009933895.1*
		GCF_900063315.1	*GCF_009933915.1*
		GCF_900063325.1	*GCF_009933935.1*
		GCF_900063335.1	*GCF_009933945.1*
		GCF_900063345.1	*GCF_009933955.1*
		GCF_900063945.1	*GCF_009933995.1*

		GCF_900063955.1	*GCF_009934015.1*
		GCF_900064045.1	*GCF_009934035.1*
		GCF_900064215.1	*GCF_009934045.1*
		GCF_900064225.1	*GCF_009934075.1*
		GCF_900064235.1	*GCF_009934095.1*
		GCF_900064245.1	*GCF_009934115.1*
		GCF_900064435.1	*GCF_009934135.1*
		GCF_900064445.1	*GCF_009934155.1*
		GCF_900064455.1	*GCF_009934175.1*
		GCF_900064505.1	*GCF_009934195.1*
		GCF_900064515.1	*GCF_009934215.1*
		GCF_900064525.1	*GCF_009934235.1*
		GCF_900064535.1	*GCF_009934255.1*
		GCF_900064545.1	*GCF_009934265.1*
		GCF_900064555.1	*GCF_009934295.1*
		GCF_900064565.1	*GCF_009934315.1*
		GCF_900064575.1	*GCF_009934335.1*
		GCF_900064585.1	*GCF_009934355.1*
		GCF_900064785.1	*GCF_009934375.1*
		GCF_900064795.1	*GCF_009934385.1*
		GCF_900065125.1	*GCF_009934395.1*
		GCF_900065325.1	*GCF_009934435.1*
		GCF_900065335.1	*GCF_009934455.1*
		GCF_900065345.1	*GCF_009934475.1*
		GCF_900065455.1	*GCF_009934485.1*
		GCF_900067275.1	*GCF_009934515.1*
		GCF_900070215.1	*GCF_009934525.1*
		GCF_900070225.1	*GCF_009934555.1*
		GCF_900070235.1	*GCF_009934575.1*
		GCF_900070245.1	*GCF_009934595.1*
		GCF_900070255.1	*GCF_009934615.1*
		GCF_900070265.1	*GCF_009934635.1*
		GCF_900070275.1	*GCF_009934655.1*
		GCF_900070285.1	*GCF_009934675.1*
		GCF_900070295.1	*GCF_009934695.1*
		GCF_900070305.1	*GCF_009934715.1*
		GCF_900080255.1	*GCF_009934735.1*
		GCF_900080265.1	*GCF_009934755.1*
		GCF_900080275.1	*GCF_009934775.1*

		GCF_900080285.1	*GCF_009934795.1*
		GCF_900080295.1	*GCF_009934815.1*
		GCF_900080305.1	*GCF_009934835.1*
		GCF_900080315.1	*GCF_009934855.1*
		GCF_900080325.1	*GCF_009934875.1*
		GCF_900080335.1	*GCF_009934895.1*
		GCF_900080345.1	*GCF_009934905.1*
		GCF_900080355.1	*GCF_009934935.1*
		GCF_900080365.1	*GCF_009934955.1*
		GCF_900080375.1	*GCF_009934975.1*
		GCF_900080385.1	*GCF_009934995.1*
		GCF_900080395.1	*GCF_009935005.1*
		GCF_900080405.1	*GCF_009935025.1*
		GCF_900080415.1	*GCF_009935055.1*
		GCF_900080425.1	*GCF_009935075.1*
		GCF_900080435.1	*GCF_009935085.1*
		GCF_900080445.1	*GCF_009935115.1*
		GCF_900080455.1	*GCF_009935185.1*
		GCF_900080465.1	*GCF_009935205.1*
		GCF_900080475.1	*GCF_011319325.1*
		GCF_900080485.1	*GCF_011319395.1*
		GCF_900080495.1	*GCF_011319405.1*
		GCF_900080505.1	*GCF_011326715.1*
		GCF_900080515.1	*GCF_011326795.1*
		GCF_900080525.1	*GCF_011326815.1*
		GCF_900080535.1	*GCF_011326825.1*
		GCF_900080545.1	*GCF_011326835.1*
		GCF_900080555.1	*GCF_011326875.1*
		GCF_900080565.1	*GCF_011326895.1*
		GCF_900080575.1	*GCF_011326915.1*
		GCF_900080585.1	*GCF_011326935.1*
		GCF_900080595.1	*GCF_011326975.1*
		GCF_900080605.1	*GCF_011326995.1*
		GCF_900080615.1	*GCF_011327005.1*
		GCF_900080625.1	*GCF_011327035.1*
		GCF_900080635.1	*GCF_011327055.1*
		GCF_900080645.1	*GCF_011327075.1*
		GCF_900080655.1	*GCF_011327085.1*
		GCF_900080665.1	*GCF_011327115.1*

		GCF_900080675.1	*GCF_011327135.1*
		GCF_900080685.1	*GCF_011327155.1*
		GCF_900080705.1	*GCF_011327175.1*
		GCF_900080715.1	*GCF_011327195.1*
		GCF_900080725.1	*GCF_011327205.1*
		GCF_900080735.1	*GCF_011327225.1*
		GCF_900080745.1	*GCF_011327235.1*
		GCF_900080755.1	*GCF_011327275.1*
		GCF_900080765.1	*GCF_011327295.1*
		GCF_900080775.1	*GCF_011327315.1*
		GCF_900080785.1	*GCF_011379065.1*
		GCF_900080795.1	*GCF_011379075.1*
		GCF_900080805.1	*GCF_011379095.1*
		GCF_900080815.1	*GCF_011379115.1*
		GCF_900080825.1	*GCF_011379165.1*
		GCF_900080835.1	*GCF_011379175.1*
		GCF_900080845.1	*GCF_011379195.1*
		GCF_900080855.1	*GCF_011379215.1*
		GCF_900080865.1	*GCF_011379245.1*
		GCF_900080875.1	*GCF_011379265.1*
		GCF_900080885.1	*GCF_011379285.1*
		GCF_900080895.1	*GCF_011379305.1*
		GCF_900080905.1	*GCF_011379325.1*
		GCF_900080915.1	*GCF_011379335.1*
		GCF_900080925.1	*GCF_011379355.1*
		GCF_900080935.1	*GCF_011379385.1*
		GCF_900080945.1	*GCF_011379405.1*
		GCF_900080955.1	*GCF_011379415.1*
		GCF_900080965.1	*GCF_011379435.1*
		GCF_900080975.1	*GCF_011379455.1*
		GCF_900080985.1	*GCF_011379465.1*
		GCF_900080995.1	*GCF_011379505.1*
		GCF_900081005.1	*GCF_011380055.1*
		GCF_900081015.1	*GCF_011383065.1*
		GCF_900081025.1	*GCF_011682135.1*
		GCF_900081035.1	*GCF_012030185.1*
		GCF_900081045.1	*GCF_012222485.1*
		GCF_900081055.1	*GCF_012593885.1*
		GCF_900081065.1	*GCF_013000945.1*

		GCF_900081075.1	*GCF_013325805.1*
		GCF_900081085.1	*GCF_013325855.1*
		GCF_900081095.1	*GCF_013328665.1*
		GCF_900081105.1	*GCF_013328715.1*
		GCF_900081115.1	*GCF_013373665.1*
		GCF_900081125.1	*GCF_013414325.1*
		GCF_900081135.1	*GCF_013786965.1*
		GCF_900081145.1	*GCF_014218075.1*
		GCF_900081155.1	*GCF_014218095.1*
		GCF_900081165.1	*GCF_014218115.1*
		GCF_900081175.1	*GCF_014218135.1*
		GCF_900081185.1	*GCF_014218155.1*
		GCF_900081195.1	*GCF_014338575.1*
		GCF_900081205.1	*GCF_014874835.1*
		GCF_900081215.1	*GCF_014874845.1*
		GCF_900081225.1	*GCF_014874885.1*
		GCF_900081235.1	*GCF_014874895.1*
		GCF_900081245.1	*GCF_014874935.1*
		GCF_900081255.1	*GCF_014874945.1*
		GCF_900081265.1	*GCF_014874975.1*
		GCF_900081275.1	*GCF_014874995.1*
		GCF_900081285.1	*GCF_014875015.1*
		GCF_900081295.1	*GCF_014875025.1*
		GCF_900081305.1	*GCF_014875035.1*
		GCF_900081315.1	*GCF_014875075.1*
		GCF_900081325.1	*GCF_014875085.1*
		GCF_900081335.1	*GCF_014875115.1*
		GCF_900081345.1	*GCF_014875125.1*
		GCF_900081355.1	*GCF_014875145.1*
		GCF_900081365.1	*GCF_014875175.1*
		GCF_900081375.1	*GCF_014875195.1*
		GCF_900081385.1	*GCF_014875205.1*
		GCF_900081395.1	*GCF_014875215.1*
		GCF_900081405.1	*GCF_014875255.1*
		GCF_900081415.1	*GCF_014875275.1*
		GCF_900081425.1	*GCF_014875295.1*
		GCF_900081435.1	*GCF_014875305.1*
		GCF_900081445.1	*GCF_014875335.1*
		GCF_900081455.1	*GCF_014875345.1*

		GCF_900081465.1	*GCF_014875375.1*
		GCF_900081475.1	*GCF_014875395.1*
		GCF_900081485.1	*GCF_014875415.1*
		GCF_900081495.1	*GCF_014875425.1*
		GCF_900081505.1	*GCF_014875455.1*
		GCF_900081515.1	*GCF_014875465.1*
		GCF_900081525.1	*GCF_014875475.1*
		GCF_900081535.1	*GCF_014875515.1*
		GCF_900081545.1	*GCF_014875535.1*
		GCF_900081555.1	*GCF_014875555.1*
		GCF_900081565.1	*GCF_014875575.1*
		GCF_900081575.1	*GCF_014875595.1*
		GCF_900081585.1	*GCF_014875605.1*
		GCF_900081595.1	*GCF_014875615.1*
		GCF_900081605.1	*GCF_014875655.1*
		GCF_900081615.1	*GCF_014875665.1*
		GCF_900081625.1	*GCF_014875695.1*
		GCF_900081635.1	*GCF_014875705.1*
		GCF_900081645.1	*GCF_014895495.1*
		GCF_900081655.1	*GCF_015221735.2*
		GCF_900081665.1	*GCF_015548515.1*
		GCF_900081675.1	*GCF_015556005.1*
		GCF_900081685.1	*GCF_016454885.1*
		GCF_900081695.1	*GCF_016454945.1*
		GCF_900081705.1	*GCF_017897765.1*
		GCF_900081715.1	*GCF_017897805.1*
		GCF_900081725.1	*GCF_017897825.1*
		GCF_900081735.1	*GCF_017897845.1*
		GCF_900081745.1	*GCF_017897865.1*
		GCF_900081755.1	*GCF_017897875.1*
		GCF_900081765.1	*GCF_017897885.1*
		GCF_900081775.1	*GCF_017897925.1*
		GCF_900081785.1	*GCF_017942325.1*
		GCF_900081795.1	*GCF_019038375.1*
		GCF_900081805.1	*GCF_019552345.1*
		GCF_900081815.1	*GCF_019793945.1*
		GCF_900081825.1	*GCF_019793995.1*
		GCF_900081835.1	*GCF_019794015.1*
		GCF_900081845.1	*GCF_019794035.1*

		GCF_900081855.1	*GCF_019794045.1*
		GCF_900081865.1	*GCF_019794075.1*
		GCF_900081875.1	*GCF_019794085.1*
		GCF_900081885.1	*GCF_019794115.1*
		GCF_900081895.1	*GCF_019794135.1*
		GCF_900081905.1	*GCF_019794635.1*
		GCF_900081915.1	*GCF_019794875.1*
		GCF_900081925.1	*GCF_020111435.1*
		GCF_900081935.1	*GCF_020111445.1*
		GCF_900081945.1	*GCF_020111455.1*
		GCF_900081955.1	*GCF_020111495.1*
		GCF_900081965.1	*GCF_020111505.1*
		GCF_900081975.1	*GCF_020111535.1*
		GCF_900081985.1	*GCF_020111555.1*
		GCF_900081995.1	*GCF_020111565.1*
		GCF_900082005.1	*GCF_020179655.1*
		GCF_900082015.1	*GCF_020179695.1*
		GCF_900082025.1	*GCF_020179715.1*
		GCF_900082035.1	*GCF_020179735.1*
		GCF_900082045.1	*GCF_020179765.1*
		GCF_900082055.1	*GCF_020179785.1*
		GCF_900082065.1	*GCF_020297185.1*
		GCF_900082075.1	*GCF_020297195.1*
		GCF_900082085.1	*GCF_020297245.1*
		GCF_900082095.1	*GCF_020297305.1*
		GCF_900082105.1	*GCF_020297345.1*
		GCF_900082115.1	*GCF_020786455.1*
		GCF_900082125.1	*GCF_020786475.1*
		GCF_900082135.1	*GCF_020786485.1*
		GCF_900082145.1	*GCF_020786535.1*
		GCF_900082155.1	*GCF_020786555.1*
		GCF_900082165.1	*GCF_020786565.1*
		GCF_900082175.1	*GCF_020786575.1*
		GCF_900082185.1	*GCF_020786615.1*
		GCF_900082195.1	*GCF_020786655.1*
		GCF_900082205.1	*GCF_020786675.1*
		GCF_900082215.1	*GCF_020786685.1*
		GCF_900082225.1	*GCF_020786715.1*
		GCF_900082235.1	*GCF_020786725.1*

		GCF_900082245.1	*GCF_020786755.1*
		GCF_900082255.1	*GCF_020786775.1*
		GCF_900082265.1	*GCF_020859495.1*
		GCF_900082275.1	*GCF_020859635.1*
		GCF_900082285.1	*GCF_021460175.1*
		GCF_900082295.1	*GCF_021460195.1*
		GCF_900082305.1	*GCF_021461105.1*
		GCF_900082315.1	*GCF_021461145.1*
		GCF_900082325.1	*GCF_021496805.1*
		GCF_900082335.1	*GCF_021496825.1*
		GCF_900082345.1	*GCF_021496845.1*
		GCF_900082355.1	*GCF_021496865.1*
		GCF_900082365.1	*GCF_021496885.1*
		GCF_900082375.1	*GCF_021496905.1*
		GCF_900082385.1	*GCF_021496945.1*
		GCF_900082395.1	*GCF_021496965.1*
		GCF_900082405.1	*GCF_021655535.1*
		GCF_900082415.1	*GCF_022585975.1*
		GCF_900082425.1	*GCF_022585995.1*
		GCF_900082435.1	*GCF_022586015.1*
		GCF_900082445.1	*GCF_022586025.1*
		GCF_900082455.1	*GCF_022586035.1*
		GCF_900082465.1	*GCF_022586055.1*
		GCF_900082475.1	*GCF_022586095.1*
		GCF_900082485.1	*GCF_022586115.1*
		GCF_900082495.1	*GCF_022586135.1*
		GCF_900082505.1	*GCF_022586155.1*
		GCF_900082515.1	*GCF_022586165.1*
		GCF_900082525.1	*GCF_022586195.1*
		GCF_900082535.1	*GCF_022586275.1*
		GCF_900082545.1	*GCF_022808295.1*
		GCF_900082555.1	*GCF_022808355.1*
		GCF_900082565.1	*GCF_022808375.1*
		GCF_900082575.1	*GCF_023145735.1*
		GCF_900082585.1	*GCF_023145745.1*
		GCF_900082595.1	*GCF_023145755.1*
		GCF_900082605.1	*GCF_023145795.1*
		GCF_900082615.1	*GCF_023145815.1*
		GCF_900082625.1	*GCF_023149175.1*

		GCF_900082635.1	*GCF_023149235.1*
		GCF_900082645.1	*GCF_023149245.1*
		GCF_900082655.1	*GCF_023149275.1*
		GCF_900082665.1	*GCF_023149295.1*
		GCF_900082675.1	*GCF_023149315.1*
		GCF_900082685.1	*GCF_023149325.1*
		GCF_900082695.1	*GCF_023149375.1*
		GCF_900082705.1	*GCF_023149385.1*
		GCF_900082715.1	*GCF_023149415.1*
		GCF_900082725.1	*GCF_023149425.1*
		GCF_900082735.1	*GCF_023149475.1*
		GCF_900082745.1	*GCF_023149495.1*
		GCF_900082755.1	*GCF_023149515.1*
		GCF_900082765.1	*GCF_023149525.1*
		GCF_900082775.1	*GCF_023149575.1*
		GCF_900082785.1	*GCF_023149635.1*
		GCF_900082795.1	*GCF_023149675.1*
		GCF_900082805.1	*GCF_023149715.1*
		GCF_900082815.1	*GCF_023149725.1*
		GCF_900082825.1	*GCF_023149865.1*
		GCF_900082835.1	*GCF_023149905.1*
		GCF_900082845.1	*GCF_023149925.1*
		GCF_900082855.1	*GCF_023149955.1*
		GCF_900082865.1	*GCF_023149975.1*
		GCF_900082875.1	*GCF_023150035.1*
		GCF_900082885.1	*GCF_023507565.1*
		GCF_900082895.1	*GCF_023507615.1*
		GCF_900082905.1	*GCF_023507635.1*
		GCF_900082915.1	*GCF_900073135.1*
		GCF_900082925.1	*GCF_900073145.1*
		GCF_900082935.1	*GCF_900073155.1*
		GCF_900082945.1	*GCF_900073165.1*
		GCF_900082955.1	*GCF_900073175.1*
		GCF_900082965.1	*GCF_900073185.1*
		GCF_900082975.1	*GCF_900073195.1*
		GCF_900082985.1	*GCF_900073205.1*
		GCF_900082995.1	*GCF_900073215.1*
		GCF_900083005.1	*GCF_900073225.1*
		GCF_900083015.1	*GCF_900073235.1*

		GCF_900083025.1	*GCF_900073245.1*
		GCF_900083035.1	*GCF_900073265.1*
		GCF_900083045.1	*GCF_900073275.1*
		GCF_900083055.1	*GCF_900073285.1*
		GCF_900083065.1	*GCF_900073295.1*
		GCF_900083075.1	*GCF_900073305.1*
		GCF_900083085.1	*GCF_900073315.1*
		GCF_900083095.1	*GCF_900073325.1*
		GCF_900083105.1	*GCF_900073335.1*
		GCF_900083115.1	*GCF_900073345.1*
		GCF_900083125.1	*GCF_900073355.1*
		GCF_900083135.1	*GCF_900073365.1*
		GCF_900083145.1	*GCF_900073375.1*
		GCF_900083155.1	*GCF_900073385.1*
		GCF_900083165.1	*GCF_900073395.1*
		GCF_900083175.1	*GCF_900073405.1*
		GCF_900083185.1	*GCF_900073415.1*
		GCF_900083195.1	*GCF_900073425.1*
		GCF_900083205.1	*GCF_900073435.1*
		GCF_900083215.1	*GCF_900073445.1*
		GCF_900083225.1	*GCF_900073455.1*
		GCF_900083235.1	*GCF_900073465.1*
		GCF_900083245.1	*GCF_900073475.1*
		GCF_900083255.1	*GCF_900073485.1*
		GCF_900083265.1	*GCF_900073495.1*
		GCF_900083275.1	*GCF_900073505.1*
		GCF_900083285.1	*GCF_900073515.1*
		GCF_900083295.1	*GCF_900073525.1*
		GCF_900083305.1	*GCF_900073535.1*
		GCF_900083315.1	*GCF_900073545.1*
		GCF_900083325.1	*GCF_900073555.1*
		GCF_900083335.1	*GCF_900073565.1*
		GCF_900083345.1	*GCF_900073575.1*
		GCF_900083355.1	*GCF_900073585.1*
		GCF_900083365.1	*GCF_900073595.1*
		GCF_900083375.1	*GCF_900073605.1*
		GCF_900083385.1	*GCF_900073615.1*
		GCF_900083395.1	*GCF_900073625.1*
		GCF_900083405.1	*GCF_900073635.1*

		GCF_900083415.1	*GCF_900073645.1*
		GCF_900083425.1	*GCF_900073655.1*
		GCF_900083435.1	*GCF_900073665.1*
		GCF_900083445.1	*GCF_900073675.1*
		GCF_900083455.1	*GCF_900073685.1*
		GCF_900083465.1	*GCF_900073695.1*
		GCF_900083475.1	*GCF_900073705.1*
		GCF_900083485.1	*GCF_900073715.1*
		GCF_900083495.1	*GCF_900073725.1*
		GCF_900083505.1	*GCF_900073735.1*
		GCF_900088495.1	*GCF_900073745.1*
		GCF_900088505.1	*GCF_900073755.1*
		GCF_900088515.1	*GCF_900073765.1*
		GCF_900092595.1	*GCF_900073775.1*
		GCF_900096745.1	*GCF_900073785.1*
		GCF_900097275.1	*GCF_900073795.1*
		GCF_900097285.1	*GCF_900073805.1*
		GCF_900097295.1	*GCF_900073815.1*
		GCF_900097305.1	*GCF_900073825.1*
		GCF_900097315.1	*GCF_900073835.1*
		GCF_900097325.1	*GCF_900073845.1*
		GCF_900097335.1	*GCF_900073855.1*
		GCF_900097345.1	*GCF_900073865.1*
		GCF_900097355.1	*GCF_900073875.1*
		GCF_900097365.1	*GCF_900073885.1*
		GCF_900097375.1	*GCF_900073895.1*
		GCF_900097385.1	*GCF_900073905.1*
		GCF_900097395.1	*GCF_900073915.1*
		GCF_900097405.1	*GCF_900073925.1*
		GCF_900097415.1	*GCF_900073935.1*
		GCF_900097425.1	*GCF_900073945.1*
		GCF_900097435.1	*GCF_900073955.1*
		GCF_900097445.1	*GCF_900073965.1*
		GCF_900097455.1	*GCF_900073975.1*
		GCF_900097465.1	*GCF_900073985.1*
		GCF_900097475.1	*GCF_900073995.1*
		GCF_900097485.1	*GCF_900074005.1*
		GCF_900097495.1	*GCF_900074015.1*
		GCF_900097505.1	*GCF_900074025.1*

		GCF_900097515.1	*GCF_900074035.1*
		GCF_900097525.1	*GCF_900074045.1*
		GCF_900097535.1	*GCF_900074055.1*
		GCF_900097545.1	*GCF_900074065.1*
		GCF_900097555.1	*GCF_900074075.1*
		GCF_900097565.1	*GCF_900074085.1*
		GCF_900097575.1	*GCF_900074095.1*
		GCF_900097585.1	*GCF_900074105.1*
		GCF_900097595.1	*GCF_900074115.1*
		GCF_900097605.1	*GCF_900074125.1*
		GCF_900097615.1	*GCF_900074135.1*
		GCF_900097625.1	*GCF_900074145.1*
		GCF_900097635.1	*GCF_900074155.1*
		GCF_900097645.1	*GCF_900074165.1*
		GCF_900097655.1	*GCF_900074175.1*
		GCF_900097665.1	*GCF_900074185.1*
		GCF_900097675.1	*GCF_900074195.1*
		GCF_900097685.1	*GCF_900074205.1*
		GCF_900097695.1	*GCF_900074215.1*
		GCF_900097705.1	*GCF_900074225.1*
		GCF_900097715.1	*GCF_900074235.1*
		GCF_900097725.1	*GCF_900074245.1*
		GCF_900097735.1	*GCF_900074255.1*
		GCF_900097745.1	*GCF_900074265.1*
		GCF_900097755.1	*GCF_900074275.1*
		GCF_900097765.1	*GCF_900074285.1*
		GCF_900097775.1	*GCF_900074295.1*
		GCF_900097785.1	*GCF_900074305.1*
		GCF_900097795.1	*GCF_900074315.1*
		GCF_900097805.1	*GCF_900074325.1*
		GCF_900097815.1	*GCF_900074335.1*
		GCF_900097825.1	*GCF_900074345.1*
		GCF_900097835.1	*GCF_900074355.1*
		GCF_900097845.1	*GCF_900074365.1*
		GCF_900097855.1	*GCF_900074375.1*
		GCF_900097865.1	*GCF_900074385.1*
		GCF_900097875.1	*GCF_900074395.1*
		GCF_900097885.1	*GCF_900074405.1*
		GCF_900097895.1	*GCF_900074415.1*

		GCF_900097905.1	*GCF_900074425.1*
		GCF_900097915.1	*GCF_900074435.1*
		GCF_900097925.1	*GCF_900074445.1*
		GCF_900097935.1	*GCF_900074455.1*
		GCF_900097945.1	*GCF_900074465.1*
		GCF_900097985.1	*GCF_900074475.1*
		GCF_900098005.1	*GCF_900074485.1*
		GCF_900098015.1	*GCF_900074495.1*
		GCF_900098025.1	*GCF_900074505.1*
		GCF_900098035.1	*GCF_900074515.1*
		GCF_900098045.1	*GCF_900074525.1*
		GCF_900098055.1	*GCF_900074535.1*
		GCF_900098065.1	*GCF_900074545.1*
		GCF_900098075.1	*GCF_900074555.1*
		GCF_900098085.1	*GCF_900074565.1*
		GCF_900098095.1	*GCF_900074575.1*
		GCF_900098105.1	*GCF_900074585.1*
		GCF_900098115.1	*GCF_900074595.1*
		GCF_900098125.1	*GCF_900074605.1*
		GCF_900098135.1	*GCF_900074615.1*
		GCF_900098145.1	*GCF_900074655.1*
		GCF_900098155.1	*GCF_900078265.1*
		GCF_900098165.1	*GCF_900155855.1*
		GCF_900098175.1	*GCF_900458935.1*
		GCF_900098195.1	*GCF_900458965.1*
		GCF_900098205.1	*GCF_900458975.1*
		GCF_900098215.1	*GCF_900458985.1*
		GCF_900098225.1	*GCF_900458995.1*
		GCF_900098235.1	*GCF_900459005.1*
		GCF_900098245.1	*GCF_900459015.1*
		GCF_900098255.1	*GCF_900459055.1*
		GCF_900098265.1	*GCF_900459075.1*
		GCF_900098275.1	*GCF_900459085.1*
		GCF_900098285.1	*GCF_900459105.1*
		GCF_900098295.1	*GCF_900459155.1*
		GCF_900098305.1	*GCF_900459235.1*
		GCF_900098315.1	*GCF_900459245.1*
		GCF_900098325.1	*GCF_900459255.1*
		GCF_900098335.1	*GCF_900459285.1*

		GCF_900098345.1	GCF_900474905.1
		GCF_900098355.1	GCF_900475355.1
		GCF_900098365.1	GCF_900636375.1
		GCF_900098375.1	GCF_900638415.1
		GCF_900098385.1	GCF_900638495.1
		GCF_900098395.1	GCF_900683545.1
		GCF_900098405.1	GCF_901875485.1
		GCF_900098415.1	GCF_902386105.1
		GCF_900098425.1	
		GCF_900098435.1	
		GCF_900098445.1	
		GCF_900098455.1	
		GCF_900098465.1	
		GCF_900098475.1	
		GCF_900098485.1	
		GCF_900098495.1	
		GCF_900098505.1	
		GCF_900098515.1	
		GCF_900098525.1	
		GCF_900098535.1	
		GCF_900098545.1	
		GCF_900098555.1	
		GCF_900098565.1	
		GCF_900098575.1	
		GCF_900098585.1	
		GCF_900098595.1	
		GCF_900098605.1	
		GCF_900098625.1	
		GCF_900098635.1	
		GCF_900098645.1	
		GCF_900098665.1	
		GCF_900098685.1	
		GCF_900098695.1	
		GCF_900098705.1	
		GCF_900098715.1	
		GCF_900098725.1	
		GCF_900124655.1	
		GCF_900124665.1	
		GCF_900124675.1	

		GCF_900124685.1	
		GCF_900124695.1	
		GCF_900124705.1	
		GCF_900124715.1	
		GCF_900124725.1	
		GCF_900124735.1	
		GCF_900124745.1	
		GCF_900124755.1	
		GCF_900124765.1	
		GCF_900124775.1	
		GCF_900124785.1	
		GCF_900124795.1	
		GCF_900124805.1	
		GCF_900124815.1	
		GCF_900124825.1	
		GCF_900124835.1	
		GCF_900124845.1	
		GCF_900124855.1	
		GCF_900124865.1	
		GCF_900124875.1	
		GCF_900124885.1	
		GCF_900124895.1	
		GCF_900124905.1	
		GCF_900124915.1	
		GCF_900124925.1	
		GCF_900124935.1	
		GCF_900124945.1	
		GCF_900124955.1	
		GCF_900124965.1	
		GCF_900124975.1	
		GCF_900124985.1	
		GCF_900124995.1	
		GCF_900125005.1	
		GCF_900125015.1	
		GCF_900125025.1	
		GCF_900125035.1	
		GCF_900125045.1	
		GCF_900125055.1	
		GCF_900125065.1	

		GCF_900125075.1	
		GCF_900125085.1	
		GCF_900125095.1	
		GCF_900125105.1	
		GCF_900125115.1	
		GCF_900125125.1	
		GCF_900125135.1	
		GCF_900125145.1	
		GCF_900125155.1	
		GCF_900125165.1	
		GCF_900125175.1	
		GCF_900125185.1	
		GCF_900125195.1	
		GCF_900125205.1	
		GCF_900125215.1	
		GCF_900125225.1	
		GCF_900125235.1	
		GCF_900125245.1	
		GCF_900125255.1	
		GCF_900125265.1	
		GCF_900125275.1	
		GCF_900125285.1	
		GCF_900125295.1	
		GCF_900125305.1	
		GCF_900125315.1	
		GCF_900125325.1	
		GCF_900125335.1	
		GCF_900125345.1	
		GCF_900125355.1	
		GCF_900125365.1	
		GCF_900125375.1	
		GCF_900125385.1	
		GCF_900125395.1	
		GCF_900125405.1	
		GCF_900125415.1	
		GCF_900125425.1	
		GCF_900125435.1	
		GCF_900125445.1	
		GCF_900125455.1	

		GCF_900125465.1	
		GCF_900125475.1	
		GCF_900125485.1	
		GCF_900125495.1	
		GCF_900125505.1	
		GCF_900125515.1	
		GCF_900125525.1	
		GCF_900125535.1	
		GCF_900125545.1	
		GCF_900125555.1	
		GCF_900125565.1	
		GCF_900125575.1	
		GCF_900125585.1	
		GCF_900125595.1	
		GCF_900125605.1	
		GCF_900125615.1	
		GCF_900125625.1	
		GCF_900125635.1	
		GCF_900125645.1	
		GCF_900125655.1	
		GCF_900125665.1	
		GCF_900125675.1	
		GCF_900125685.1	
		GCF_900125695.1	
		GCF_900125705.1	
		GCF_900125715.1	
		GCF_900125725.1	
		GCF_900125735.1	
		GCF_900125745.1	
		GCF_900125755.1	
		GCF_900125765.1	
		GCF_900125775.1	
		GCF_900125785.1	
		GCF_900125795.1	
		GCF_900125805.1	
		GCF_900125815.1	
		GCF_900125825.1	
		GCF_900125835.1	
		GCF_900125845.1	

		GCF_900125855.1	
		GCF_900125865.1	
		GCF_900125875.1	
		GCF_900125885.1	
		GCF_900125895.1	
		GCF_900125905.1	
		GCF_900125915.1	
		GCF_900125925.1	
		GCF_900125935.1	
		GCF_900125945.1	
		GCF_900125955.1	
		GCF_900125965.1	
		GCF_900125975.1	
		GCF_900125985.1	
		GCF_900125995.1	
		GCF_900126005.1	
		GCF_900126015.1	
		GCF_900126025.1	
		GCF_900126035.1	
		GCF_900126045.1	
		GCF_900126055.1	
		GCF_900126065.1	
		GCF_900126075.1	
		GCF_900126085.1	
		GCF_900126095.1	
		GCF_900126105.1	
		GCF_900126115.1	
		GCF_900126125.1	
		GCF_900126135.1	
		GCF_900126145.1	
		GCF_900126155.1	
		GCF_900126165.1	
		GCF_900126175.1	
		GCF_900126185.1	
		GCF_900126195.1	
		GCF_900126205.1	
		GCF_900126215.1	
		GCF_900126225.1	
		GCF_900126235.1	

		GCF_900126245.1	
		GCF_900126255.1	
		GCF_900126265.1	
		GCF_900126275.1	
		GCF_900126285.1	
		GCF_900126295.1	
		GCF_900126305.1	
		GCF_900126315.1	
		GCF_900126325.1	
		GCF_900126335.1	
		GCF_900126345.1	
		GCF_900126355.1	
		GCF_900126365.1	
		GCF_900126375.1	
		GCF_900126385.1	
		GCF_900126395.1	
		GCF_900126405.1	
		GCF_900126415.1	
		GCF_900126425.1	
		GCF_900126435.1	
		GCF_900126445.1	
		GCF_900126455.1	
		GCF_900126465.1	
		GCF_900126475.1	
		GCF_900126485.1	
		GCF_900126495.1	
		GCF_900126505.1	
		GCF_900126515.1	
		GCF_900126535.1	
		GCF_900126575.1	
		GCF_900126635.1	
		GCF_900126715.1	
		GCF_900126755.1	
		GCF_900126765.1	
		GCF_900126845.1	
		GCF_900126925.1	
		GCF_900127015.1	
		GCF_900127095.1	
		GCF_900127105.1	

		GCF_900127635.1	
		GCF_900127645.1	
		GCF_900127655.1	
		GCF_900127665.1	
		GCF_900127685.1	
		GCF_900127695.1	
		GCF_900127705.1	
		GCF_900127715.1	
		GCF_900127725.1	
		GCF_900127735.1	
		GCF_900127745.1	
		GCF_900127755.1	
		GCF_900127765.1	
		GCF_900127775.1	
		GCF_900127785.1	
		GCF_900127795.1	
		GCF_900127805.1	
		GCF_900127815.1	
		GCF_900127825.1	
		GCF_900127835.1	
		GCF_900127845.1	
		GCF_900127855.1	
		GCF_900127865.1	
		GCF_900127875.1	
		GCF_900127885.1	
		GCF_900127895.1	
		GCF_900127905.1	
		GCF_900127915.1	
		GCF_900127925.1	
		GCF_900127935.1	
		GCF_900127945.1	
		GCF_900127955.1	
		GCF_900127965.1	
		GCF_900127975.1	
		GCF_900127985.1	
		GCF_900127995.1	
		GCF_900128005.1	
		GCF_900128015.1	
		GCF_900128035.1	

		GCF_900128045.1	
		GCF_900128055.1	
		GCF_900128065.1	
		GCF_900128075.1	
		GCF_900128085.1	
		GCF_900128095.1	
		GCF_900128105.1	
		GCF_900128115.1	
		GCF_900128125.1	
		GCF_900128135.1	
		GCF_900128285.1	
		GCF_900128295.1	
		GCF_900128305.1	
		GCF_900128315.1	
		GCF_900128375.1	
		GCF_900129335.1	
		GCF_900149245.1	
		GCF_900149255.1	
		GCF_900149265.1	
		GCF_900149275.1	
		GCF_900149325.1	
		GCF_900149335.1	
		GCF_900155705.1	
		GCF_900155715.1	
		GCF_900155725.1	
		GCF_900155745.1	
		GCF_900155755.1	
		GCF_900155765.1	
		GCF_900155775.1	
		GCF_900155785.1	
		GCF_900155795.1	
		GCF_900155805.1	
		GCF_900155815.1	
		GCF_900155825.1	
		GCF_900155835.1	
		GCF_900155845.1	
		GCF_900155875.1	
		GCF_900233935.1	
		GCF_900235965.1	

		GCF_900235975.1	
		GCF_900235985.1	
		GCF_900235995.1	
		GCF_900236005.1	
		GCF_900236025.1	
		GCF_900240125.1	
		GCF_900250155.1	
		GCF_900250165.1	
		GCF_900250175.1	
		GCF_900250185.1	
		GCF_900250195.1	
		GCF_900250205.1	
		GCF_900250215.1	
		GCF_900250225.1	
		GCF_900250235.1	
		GCF_900250245.1	
		GCF_900250255.1	
		GCF_900250265.1	
		GCF_900250275.1	
		GCF_900250285.1	
		GCF_900250295.1	
		GCF_900250305.1	
		GCF_900250315.1	
		GCF_900250325.1	
		GCF_900250335.1	
		GCF_900250345.1	
		GCF_900250355.1	
		GCF_900250365.1	
		GCF_900250375.1	
		GCF_900250385.1	
		GCF_900250395.1	
		GCF_900250405.1	
		GCF_900250415.1	
		GCF_900250425.1	
		GCF_900250435.1	
		GCF_900250445.1	
		GCF_900250455.1	
		GCF_900250465.1	
		GCF_900250475.1	

		GCF_900250485.1	
		GCF_900250495.1	
		GCF_900250505.1	
		GCF_900250515.1	
		GCF_900250525.1	
		GCF_900250535.1	
		GCF_900250545.1	
		GCF_900250555.1	
		GCF_900250565.1	
		GCF_900250575.1	
		GCF_900250585.1	
		GCF_900250595.1	
		GCF_900250605.1	
		GCF_900250615.1	
		GCF_900250625.1	
		GCF_900250635.1	
		GCF_900250645.1	
		GCF_900250655.1	
		GCF_900250665.1	
		GCF_900250675.1	
		GCF_900250685.1	
		GCF_900250695.1	
		GCF_900250705.1	
		GCF_900250715.1	
		GCF_900250725.1	
		GCF_900250735.1	
		GCF_900250745.1	
		GCF_900250755.1	
		GCF_900250765.1	
		GCF_900250775.1	
		GCF_900250785.1	
		GCF_900250795.1	
		GCF_900250805.1	
		GCF_900250815.1	
		GCF_900250825.1	
		GCF_900250835.1	
		GCF_900250845.1	
		GCF_900250855.1	
		GCF_900250865.1	

		GCF_900250875.1	
		GCF_900250885.1	
		GCF_900250895.1	
		GCF_900250905.1	
		GCF_900250915.1	
		GCF_900250925.1	
		GCF_900250935.1	
		GCF_900250945.1	
		GCF_900250955.1	
		GCF_900250965.1	
		GCF_900250975.1	
		GCF_900250985.1	
		GCF_900250995.1	
		GCF_900251005.1	
		GCF_900251015.1	
		GCF_900251025.1	
		GCF_900251035.1	
		GCF_900251045.1	
		GCF_900251055.1	
		GCF_900251065.1	
		GCF_900251075.1	
		GCF_900251085.1	
		GCF_900251095.1	
		GCF_900251105.1	
		GCF_900251115.1	
		GCF_900251125.1	
		GCF_900251135.1	
		GCF_900251145.1	
		GCF_900251155.1	
		GCF_900251165.1	
		GCF_900251175.1	
		GCF_900251185.1	
		GCF_900251195.1	
		GCF_900251205.1	
		GCF_900251215.1	
		GCF_900251225.1	
		GCF_900251235.1	
		GCF_900251245.1	
		GCF_900251255.1	

		GCF_900251265.1	
		GCF_900251275.1	
		GCF_900251285.1	
		GCF_900251295.1	
		GCF_900251305.1	
		GCF_900251315.1	
		GCF_900251325.1	
		GCF_900251335.1	
		GCF_900251345.1	
		GCF_900251355.1	
		GCF_900251365.1	
		GCF_900251375.1	
		GCF_900251385.1	
		GCF_900251395.1	
		GCF_900251405.1	
		GCF_900251415.1	
		GCF_900251425.1	
		GCF_900251435.1	
		GCF_900251445.1	
		GCF_900251455.1	
		GCF_900251465.1	
		GCF_900251475.1	
		GCF_900251485.1	
		GCF_900251495.1	
		GCF_900251505.1	
		GCF_900251515.1	
		GCF_900251525.1	
		GCF_900251535.1	
		GCF_900251545.1	
		GCF_900251555.1	
		GCF_900251565.1	
		GCF_900251575.1	
		GCF_900251585.1	
		GCF_900251595.1	
		GCF_900251605.1	
		GCF_900251615.1	
		GCF_900251625.1	
		GCF_900251635.1	
		GCF_900251645.1	

		GCF_900251655.1	
		GCF_900251665.1	
		GCF_900251675.1	
		GCF_900251685.1	
		GCF_900251695.1	
		GCF_900251705.1	
		GCF_900251715.1	
		GCF_900251725.1	
		GCF_900251735.1	
		GCF_900251745.1	
		GCF_900251755.1	
		GCF_900251765.1	
		GCF_900251775.1	
		GCF_900251785.1	
		GCF_900251795.1	
		GCF_900251805.1	
		GCF_900251815.1	
		GCF_900251825.1	
		GCF_900251835.1	
		GCF_900251845.1	
		GCF_900251855.1	
		GCF_900251865.1	
		GCF_900251875.1	
		GCF_900251885.1	
		GCF_900251895.1	
		GCF_900251905.1	
		GCF_900251915.1	
		GCF_900251925.1	
		GCF_900251935.1	
		GCF_900251945.1	
		GCF_900251955.1	
		GCF_900251965.1	
		GCF_900251975.1	
		GCF_900251985.1	
		GCF_900251995.1	
		GCF_900252005.1	
		GCF_900252015.1	
		GCF_900252025.1	
		GCF_900252035.1	

		GCF_900252045.1	
		GCF_900252055.1	
		GCF_900252065.1	
		GCF_900252075.1	
		GCF_900252085.1	
		GCF_900252095.1	
		GCF_900252105.1	
		GCF_900252115.1	
		GCF_900252125.1	
		GCF_900252135.1	
		GCF_900252145.1	
		GCF_900252155.1	
		GCF_900252165.1	
		GCF_900252175.1	
		GCF_900252185.1	
		GCF_900252195.1	
		GCF_900252205.1	
		GCF_900252215.1	
		GCF_900323905.1	
		GCF_900323925.1	
		GCF_900323955.1	
		GCF_900323965.1	
		GCF_900324045.1	
		GCF_900324065.1	
		GCF_900324205.1	
		GCF_900324215.1	
		GCF_900324225.1	
		GCF_900324235.1	
		GCF_900324245.1	
		GCF_900324255.1	
		GCF_900324265.1	
		GCF_900324275.1	
		GCF_900324285.1	
		GCF_900324295.1	
		GCF_900324305.1	
		GCF_900324315.1	
		GCF_900324325.1	
		GCF_900324335.1	
		GCF_900324345.1	

		GCF_900324355.1	
		GCF_900324365.1	
		GCF_900324385.1	
		GCF_900324405.1	
		GCF_900324415.1	
		GCF_900409475.1	
		GCF_900457275.1	
		GCF_900457285.1	
		GCF_900457295.1	
		GCF_900457315.1	
		GCF_900457325.1	
		GCF_900457345.1	
		GCF_900457385.1	
		GCF_900457395.1	
		GCF_900457405.1	
		GCF_900457415.1	
		GCF_900457445.1	
		GCF_900457455.1	
		GCF_900457485.1	
		GCF_900457505.1	
		GCF_900457525.1	
		GCF_900457545.1	
		GCF_900457555.1	
		GCF_900457565.1	
		GCF_900457575.1	
		GCF_900457585.1	
		GCF_900457605.1	
		GCF_900457625.1	
		GCF_900457635.1	
		GCF_900457645.1	
		GCF_900457665.1	
		GCF_900457675.1	
		GCF_900457685.1	
		GCF_900457695.1	
		GCF_900457715.1	
		GCF_900457725.1	
		GCF_900457745.1	
		GCF_900457755.1	
		GCF_900457765.1	

		GCF_900457775.1	
		GCF_900457785.1	
		GCF_900457795.1	
		GCF_900457805.1	
		GCF_900457825.1	
		GCF_900457835.1	
		GCF_900457845.1	
		GCF_900457855.1	
		GCF_900457865.1	
		GCF_900457875.1	
		GCF_900457885.1	
		GCF_900457895.1	
		GCF_900457905.1	
		GCF_900457925.1	
		GCF_900457935.1	
		GCF_900457955.1	
		GCF_900457965.1	
		GCF_900457975.1	
		GCF_900457995.1	
		GCF_900458005.1	
		GCF_900458025.1	
		GCF_900458035.1	
		GCF_900458045.1	
		GCF_900458055.1	
		GCF_900458065.1	
		GCF_900458075.1	
		GCF_900458085.1	
		GCF_900458095.1	
		GCF_900458105.1	
		GCF_900458115.1	
		GCF_900458145.1	
		GCF_900458155.1	
		GCF_900458165.1	
		GCF_900458175.1	
		GCF_900458185.1	
		GCF_900458225.1	
		GCF_900458235.1	
		GCF_900458265.1	
		GCF_900458275.1	

		GCF_900458295.1	
		GCF_900458305.1	
		GCF_900458315.1	
		GCF_900458325.1	
		GCF_900458335.1	
		GCF_900458345.1	
		GCF_900458395.1	
		GCF_900458405.1	
		GCF_900458415.1	
		GCF_900458445.1	
		GCF_900458455.1	
		GCF_900458465.1	
		GCF_900458475.1	
		GCF_900458485.1	
		GCF_900458495.1	
		GCF_900458725.1	
		GCF_900474525.1	
		GCF_900474535.1	
		GCF_900474555.1	
		GCF_900474565.1	
		GCF_900474575.1	
		GCF_900474665.1	
		GCF_900474675.1	
		GCF_900474695.1	
		GCF_900474715.1	
		GCF_900474725.1	
		GCF_900474735.1	
		GCF_900474755.1	
		GCF_900475055.1	
		GCF_900475245.1	
		GCF_900478245.1	
		GCF_900482515.1	
		GCF_900482525.1	
		GCF_900482535.1	
		GCF_900482555.1	
		GCF_900482565.1	
		GCF_900482595.1	
		GCF_900482605.1	
		GCF_900482615.1	

		GCF_900482625.1	
		GCF_900482645.1	
		GCF_900482655.1	
		GCF_900482665.1	
		GCF_900482675.1	
		GCF_900482685.1	
		GCF_900482695.1	
		GCF_900482705.1	
		GCF_900482725.1	
		GCF_900482735.1	
		GCF_900482745.1	
		GCF_900482755.1	
		GCF_900482765.1	
		GCF_900482775.1	
		GCF_900482785.1	
		GCF_900482795.1	
		GCF_900482805.1	
		GCF_900482815.1	
		GCF_900482825.1	
		GCF_900482835.1	
		GCF_900482845.1	
		GCF_900482855.1	
		GCF_900482865.1	
		GCF_900482875.1	
		GCF_900482885.1	
		GCF_900482895.1	
		GCF_900482905.1	
		GCF_900482915.1	
		GCF_900482925.1	
		GCF_900482935.1	
		GCF_900482945.1	
		GCF_900482955.1	
		GCF_900482965.1	
		GCF_900482975.1	
		GCF_900482985.1	
		GCF_900482995.1	
		GCF_900483005.1	
		GCF_900483015.1	
		GCF_900483025.1	

		GCF_900483035.1	
		GCF_900483045.1	
		GCF_900483055.1	
		GCF_900483065.1	
		GCF_900483075.1	
		GCF_900483085.1	
		GCF_900483095.1	
		GCF_900483105.1	
		GCF_900483115.1	
		GCF_900483125.1	
		GCF_900483135.1	
		GCF_900483145.1	
		GCF_900483155.1	
		GCF_900483165.1	
		GCF_900483175.1	
		GCF_900483185.1	
		GCF_900483195.1	
		GCF_900483205.1	
		GCF_900483215.1	
		GCF_900483225.1	
		GCF_900483235.1	
		GCF_900483245.1	
		GCF_900483255.1	
		GCF_900483265.1	
		GCF_900483275.1	
		GCF_900483285.1	
		GCF_900483295.1	
		GCF_900483305.1	
		GCF_900483315.1	
		GCF_900483325.1	
		GCF_900483335.1	
		GCF_900483345.1	
		GCF_900483355.1	
		GCF_900483365.1	
		GCF_900483375.1	
		GCF_900483385.1	
		GCF_900483395.1	
		GCF_900483405.1	
		GCF_900483415.1	

		GCF_900483425.1	
		GCF_900483435.1	
		GCF_900483445.1	
		GCF_900483455.1	
		GCF_900483465.1	
		GCF_900483475.1	
		GCF_900483485.1	
		GCF_900483495.1	
		GCF_900483505.1	
		GCF_900483515.1	
		GCF_900483525.1	
		GCF_900483535.1	
		GCF_900483545.1	
		GCF_900483555.1	
		GCF_900483565.1	
		GCF_900483575.1	
		GCF_900483585.1	
		GCF_900483595.1	
		GCF_900483605.1	
		GCF_900483615.1	
		GCF_900483625.1	
		GCF_900483635.1	
		GCF_900483645.1	
		GCF_900483655.1	
		GCF_900483665.1	
		GCF_900483675.1	
		GCF_900483685.1	
		GCF_900483695.1	
		GCF_900483705.1	
		GCF_900483715.1	
		GCF_900483725.1	
		GCF_900483735.1	
		GCF_900483745.1	
		GCF_900483755.1	
		GCF_900483765.1	
		GCF_900483775.1	
		GCF_900483785.1	
		GCF_900483795.1	
		GCF_900483805.1	

		GCF_900483815.1	
		GCF_900483825.1	
		GCF_900483835.1	
		GCF_900483845.1	
		GCF_900483855.1	
		GCF_900483865.1	
		GCF_900483875.1	
		GCF_900483885.1	
		GCF_900483895.1	
		GCF_900483905.1	
		GCF_900483915.1	
		GCF_900483925.1	
		GCF_900483935.1	
		GCF_900483945.1	
		GCF_900483955.1	
		GCF_900483965.1	
		GCF_900483975.1	
		GCF_900483985.1	
		GCF_900483995.1	
		GCF_900484005.1	
		GCF_900484015.1	
		GCF_900484025.1	
		GCF_900484035.1	
		GCF_900484045.1	
		GCF_900484055.1	
		GCF_900484065.1	
		GCF_900484075.1	
		GCF_900484085.1	
		GCF_900484095.1	
		GCF_900484105.1	
		GCF_900484115.1	
		GCF_900484125.1	
		GCF_900484135.1	
		GCF_900484145.1	
		GCF_900484155.1	
		GCF_900484165.1	
		GCF_900484175.1	
		GCF_900484185.1	
		GCF_900484225.1	

		GCF_900484235.1	
		GCF_900484255.1	
		GCF_900484295.1	
		GCF_900484305.1	
		GCF_900484325.1	
		GCF_900484365.1	
		GCF_900484395.1	
		GCF_900484405.1	
		GCF_900484425.1	
		GCF_900484495.1	
		GCF_900484515.1	
		GCF_900484535.1	
		GCF_900490315.1	
		GCF_900490335.1	
		GCF_900490345.1	
		GCF_900490425.1	
		GCF_900607245.1	
		GCF_900607255.1	
		GCF_900607265.1	
		GCF_900607275.1	
		GCF_900607285.1	
		GCF_900607295.1	
		GCF_900607305.1	
		GCF_900620215.1	
		GCF_900620225.1	
		GCF_900620235.1	
		GCF_900620245.1	
		GCF_900620255.1	
		GCF_900635095.1	
		GCF_900635245.1	
		GCF_900635265.1	
		GCF_900635285.1	
		GCF_900635305.1	
		GCF_900635315.1	
		GCF_900635335.1	
		GCF_900635505.1	
		GCF_900635905.1	
		GCF_900636335.1	
		GCF_900636695.1	

		GCF_900637155.1	
		GCF_901543185.1	
		GCF_901669825.1	
		GCF_901875495.1	
		GCF_902166055.1	
		GCF_902385895.1	
		GCF_902636275.1	
		GCF_902703405.1	
		GCF_902715065.1	
		GCF_902715085.1	
		GCF_902715105.1	
		GCF_902715115.1	
		GCF_902715265.1	
		GCF_902715275.1	
		GCF_902715285.1	
		GCF_902715315.1	
		GCF_902715325.1	
		GCF_902715335.1	
		GCF_902715345.1	
		GCF_902715365.1	
		GCF_902715375.1	
		GCF_902715385.1	
		GCF_902715395.1	
		GCF_902715405.1	
		GCF_902715415.1	
		GCF_902715425.1	
		GCF_902715435.1	
		GCF_902715455.1	
		GCF_902717235.1	
		GCF_902720405.1	
		GCF_902720795.1	
		GCF_902722345.1	
		GCF_902722395.1	
		GCF_902722465.1	
		GCF_902722515.1	
		GCF_902723625.1	
		GCF_903932595.1	
		GCF_903932605.1	
		GCF_910589445.1	

		GCF_910589465.1	
		GCF_910589495.1	
		GCF_910589585.1	
		GCF_910589705.1	
		GCF_910589785.1	
		GCF_910589795.1	
		GCF_910589815.1	
		GCF_910589825.1	
		GCF_910589845.1	
		GCF_910589855.1	
		GCF_910589865.1	
		GCF_910589885.1	
		GCF_910589895.1	
		GCF_910589915.1	
		GCF_910589965.1	
		GCF_910589975.1	
		GCF_910589985.1	
		GCF_910589995.1	
		GCF_910590005.1	
		GCF_910590015.1	
		GCF_910590025.1	
		GCF_910590095.1	
		GCF_910590105.1	
		GCF_910590135.1	
		GCF_910590145.1	
		GCF_910590175.1	
		GCF_910590185.1	
		GCF_910590205.1	
		GCF_918417985.1	
		GCF_918418275.1	
		GCF_918419285.1	
		GCF_918422865.1	
		GCF_918429095.1	
		GCF_918429155.1	
		GCF_933208685.1	
		GCF_933208905.1	
		GCF_933217055.1	
		GCF_933217245.1	
		GCF_933217265.1	

		GCF_933217415.1	
		GCF_933217435.1	
		GCF_933217555.1	
		GCF_933217595.1	
		GCF_933217655.1	
		GCF_936275985.1	
		GCF_936384895.1	
		GCF_936394065.1	

Table S2. The analysis of affinity primers in single PCR and multiplex PCR were calculated by Primer-Dimer software.

Single PCR primer-primer interaction

Forward Primer Name	Reverse Primer Name	Structure	dG [kcal/mole]
Sraga_1_F	Sraga_1_R	heterodimer	-3,58
Srdys_1_F	Srdys_1_R	heterodimer	-0,08
Srube_3_F	Srube_3_R	heterodimer	0
Staur_3_F	Staur_3_R	heterodimer	-1,8
**Multiplex PCR primer-primer interaction**			
Sraga_1_F	Sraga_1_R	heterodimer	-3,58
Sraga_1_F	Srdys_1_F	heterodimer	-2
Sraga_1_F	Srdys_1_R	heterodimer	0
Sraga_1_F	Srube_3_F	heterodimer	-0,06
Sraga_1_F	Srube_3_R	heterodimer	-1,31
Sraga_1_F	Staur_3_F	heterodimer	-2,11
Sraga_1_F	Staur_3_R	heterodimer	-0,05
Sraga_1_R	Srdys_1_F	heterodimer	-3,58
Sraga_1_R	Srdys_1_R	heterodimer	-3,58
Sraga_1_R	Srube_3_F	heterodimer	-3,58
Sraga_1_R	Srube_3_R	heterodimer	-3,58
Sraga_1_R	Staur_3_F	heterodimer	-3,58
Sraga_1_R	Staur_3_R	heterodimer	-3,58
Srdys_1_F	Srdys_1_R	heterodimer	-0,08
Srdys_1_F	Srube_3_F	heterodimer	-0,08
Srdys_1_F	Srube_3_R	heterodimer	-0,08
Srdys_1_F	Staur_3_F	heterodimer	-1,8
Srdys_1_F	Staur_3_R	heterodimer	-0,08
Srdys_1_R	Srube_3_F	heterodimer	-2,18
Srdys_1_R	Srube_3_R	heterodimer	-2,33
Srdys_1_R	Staur_3_F	heterodimer	-2,37
Srdys_1_R	Staur_3_R	heterodimer	0
Srube_3_F	Srube_3_R	heterodimer	0

Srube_3_F	Staur_3_F	heterodimer	-1,8
Srube_3_F	Staur_3_R	heterodimer	-0,4
Srube_3_R	Staur_3_F	heterodimer	-1,8
Srube_3_R	Staur_3_R	heterodimer	-2,39
Staur_3_F	Staur_3_R	heterodimer	-1,8

Table S3. The statistics of designed PCR tests.

Species	*S. agalactiae*	*S. dysgalactiae*	*S. uberis*	*S. aureus*
**Primers**	Sraga_1	Srdys_1	Srube_3	Staur_3
**Condition positive (P)**	1466	132	108	14204
**Condition negative (N)**	15153	16487	16511	4616
**True positive (TP)**	1466	132	108	14193
**True negative (TN)**	15123	16484	16511	4562
**False positive (FP), Type I error**	30	3	0	53
**False negative (FN), Type II error**	0	0	0	12
**Sensitivity** $(\frac{TP}{TP+FN})$	1	1	1	0.9992
**Specificity** $\frac{TN}{TN+FP})$	0.9980	0.9998	1	0.9883
**Precision** $(\frac{TP}{TP+FP})$	0.9799	0.9778	1	0.9963
**Prevalence** $(\frac{P}{P+N})$	0.0882	0.0079	0.0065	0.7547
**Accuracy** $(\frac{TP+TN}{P+N})$	0.9982	0.9998	1	0.9965

Table S4. Characteristics of strains used in the study.

Strain	Taxonomy reclassification after bioinformatics analysis	Animal species	Origin
S. warneri ATCC 27836	*n/a*	Human	no info
A. viridans ATCC 11563	*n/a*	no info	Environmental
E. faecalis ATCC 29212	*n/a*	no info	no info
S. aureus ATCC 6538P	-	no info	no info
S. aureus PCM 1102	-	Human	Clinical material
S. aureus PCM 1115	-	Human	Clinical material
S. aureus PCM 1116	-	Human	Clinical material
S. aureus PCM 1650	-	Human	Clinical material
S. aureus PCM 1937	-	Human	Clinical material
S. aureus PCM 2054	-	no info	no info
S. aureus PCM 2101	-	no info	no info
S. aureus PCM 2267	-	no info	no info
S. aureus PCM 458/2195	-	no info	no info
S. aureus PCM 502	-	Human	Pleural fluid
S. aureus PCM 565	-	Human	Empyema
S. epidermidis ATCC 14990	*n/a*	no info	no info
A. viridans 007PP2021	*A. urinaeequi* 002PP2021	Dairy cow	Udder milk
A. viridans 008PP2021	*M. sciuri* 004PP2021	Dairy cow	Udder milk
E. faecalis 022PP2021	-	Dairy cow	Udder milk
E. faecium 003PP2021	-	Dairy cow	Udder milk
E. faecium 004PP2021	-	Dairy cow	Udder milk
Enterococcus sp. 009PP2022	*n/a*	Dairy cow	Udder milk
Enterococcus sp. 010PP2022	*n/a*	Dairy cow	Udder milk

Strain	Taxonomy reclassification after bioinformatics analysis	Animal species	Origin
Enterococcus sp. 011PP2022	*n/a*	Dairy cow	Udder milk
Enterococcus sp. 012PP2022	*n/a*	Dairy cow	Udder milk
Enterococcus sp. 013PP2022	*n/a*	Dairy cow	Udder milk
S. agalactiae 021PP2018	*n/a*	Dairy cow	Udder milk
S. agalactiae 022PP2021	*n/a*	Dairy cow	Udder milk
S. agalactiae 023PP2021	-	Dairy cow	Udder milk
S. agalactiae 024PP2021	-	Dairy cow	Udder milk
S. agalactiae 025PP2021	*S. dysgalactiae* 004PP2021	Dairy cow	Udder milk
S. agalactiae 026PP2021	*S. dysgalactiae* 005PP2021	Dairy cow	Udder milk
S. agalactiae 027PP2021	-	Dairy cow	Udder milk
S. dysgalactiae 001PP2016	*n/a*	Dairy cow	Udder milk
S. dysgalactiae 002PP2016	*E. casseliflavus* 002PP2016	Dairy cow	Udder milk
S. dysgalactiae 003PP2016	*S. uberis* 120PP2022	Dairy cow	Udder milk
S. epidermidis 001PP2022	*S. haemolyticus* 004PP2022	Swine	Skin wound
S. sciuri 001PP2020	-	Dairy cow	Udder milk
S. sciuri 002PP2021	*S. agalactiae* 028PP2022	Dairy cow	Udder milk
S. sciuri 003PP2021	*S. equorum* 003PP2022	Dairy cow	Udder milk
S. uberis 041PP2021	-	Dairy cow	Udder milk
S. uberis 045PP2021	-	Dairy cow	Udder milk
S. uberis 059PP2021	-	Dairy cow	Udder milk
S. uberis 067PP2021	-	Dairy cow	Udder milk
S. uberis 071PP2021	-	Dairy cow	Udder milk
S. uberis 103PP2021	-	Dairy cow	Udder milk
S. uberis 105PP2021	-	Dairy cow	Udder milk
S. uberis 112PP2021	-	Dairy cow	Udder milk
S. xylosus 001PP2021	*S. equorum* 001PP2022	Dairy cow	Udder milk
S. xylosus 002PP2022	*S. equorum* 002PP2022	Dairy cow	Udder milk
S. xylosus 003PP2022	*M. vitulinus* 001PP2022	Dairy cow	Udder milk
Staphylococcus sp. 008PP2022	*n/a*	Dairy cow	Udder milk
Staphylococcus sp. 009PP2022	*n/a*	Dairy cow	Udder milk
Staphylococcus sp. 010PP2022	*n/a*	Dairy cow	Udder milk
Staphylococcus sp. 011PP2022	*n/a*	Dairy cow	Udder milk
Staphylococcus sp. 012PP2022	*n/a*	Dairy cow	Udder milk
Staphylococcus sp. 013PP2022	*n/a*	Dairy cow	Udder milk
Staphylococcus sp. 014PP2022	*n/a*	Dairy cow	Udder milk
Staphylococcus sp. 015PP2022	*n/a*	Dairy cow	Udder milk
Staphylococcus sp. 016PP2022	*n/a*	Dairy cow	Udder milk
Staphylococcus sp. 017PP2022	*n/a*	Dairy cow	Udder milk
Staphylococcus sp. 018PP2022	*n/a*	Dairy cow	Udder milk
Staphylococcus sp. 019PP2022	*n/a*	Dairy cow	Udder milk
Staphylococcus sp. 023PP2022	*n/a*	Swine	Skin wound
Staphylococcus sp. 025PP2022	*n/a*	Dairy cow	Udder milk
Staphylococcus sp. 026PP2022	*n/a*	Dairy cow	Udder milk
Staphylococcus sp. 027PP2022	*n/a*	Dairy cow	Udder milk
Staphylococcus sp. 030PP2022	*n/a*	Dairy cow	Udder milk
Staphylococcus sp. 031PP2022	*n/a*	Dairy cow	Udder milk
Staphylococcus sp. 032PP2022	*n/a*	Dairy cow	Udder milk
Staphylococcus sp. 034PP2022	*n/a*	Dairy cow	Udder milk
Staphylococcus sp. 038PP2022	*n/a*	Dairy cow	Udder milk
Staphylococcus sp. 039PP2022	*n/a*	Dairy cow	Udder milk
Staphylococcus sp. 040PP2022	*n/a*	Dairy cow	Udder milk
Staphylococcus sp. 041PP2022	*n/a*	Dairy cow	Udder milk
Staphylococcus sp. 042PP2022	*n/a*	Dairy cow	Udder milk
Staphylococcus sp. 043PP2022	*n/a*	Dairy cow	Udder milk

Strain	Taxonomy reclassification after bioinformatics analysis	Animal species	Origin
Staphylococcus sp. 044PP2022	*n/a*	Dairy cow	Udder milk
Staphylococcus sp. 045PP2022	*n/a*	Dairy cow	Udder milk
Staphylococcus sp. 046PP2022	*n/a*	Dairy cow	Udder milk
Staphylococcus sp. 047PP2022	*S. pseudintermedius* 001PP2023	Dog	Urine
Staphylococcus sp. 048PP2022	*S. pseudintermedius* 002PP2023	Dog	Eye
Staphylococcus sp. 049PP2022	*S. simulans* 002PP2022	Cat	Throat
Staphylococcus sp. 050PP2022	*S. pseudintermedius* 003PP2023	Dog	Ear
Staphylococcus sp. 051PP2022	*S. pseudintermedius* 004PP2023	Dog	Vagina
Staphylococcus sp. 052PP2022	*S. pseudintermedius* 005PP2023	Dog	Vagina
Staphylococcus sp. 053PP2022	*S. pseudintermedius* 006PP2023	Dog	Vagina
Staphylococcus sp. 054PP2022	*S. pseudintermedius* 007PP2023	Dog	Urine
Staphylococcus sp. 055PP2022	*S. pseudintermedius* 011PP2023	Cat	Urine
Staphylococcus sp. 056PP2022	*S. pseudintermedius* 012PP2023	Dog	Skin
Staphylococcus sp. 057PP2022	*n/a*	Dog	Wound
Staphylococcus sp. 059PP2022	*M. sciuri* 005PP2023	Dog	Larynx
Streptococcus sp. 003PP2017	*n/a*	Dairy cow	Udder milk
Streptococcus sp. 004PP2017	*n/a*	Dairy cow	Udder milk
Streptococcus sp. 006PP2017	*n/a*	Dairy cow	Udder milk
Streptococcus sp. 008PP2018	*n/a*	Dairy cow	Udder milk
Streptococcus sp. 009PP2019	*n/a*	Dairy cow	Udder milk
Streptococcus sp. 014PP2022	*n/a*	Dairy cow	Udder milk
Streptococcus sp. 017PP2022	*n/a*	Dairy cow	Udder milk
Streptococcus sp. 018PP2022	*n/a*	Dairy cow	Udder milk
NTC – No Template Control			

n/a – not applicable, strain was not sequenced; dash - no change in taxonomy after sequencing
